# Reconstructing Spatial Localization Error Maps via Physics-Informed Tensor Completion for Passive Sensor Systems

**DOI:** 10.3390/s26020597

**Published:** 2026-01-15

**Authors:** Zhaohang Zhang, Zhen Huang, Chunzhe Wang, Qiaowen Jiang

**Affiliations:** 1 Department of Electronic Engineering, Tsinghua University, Beijing 100084, China; zhangzh22@mails.tsinghua.edu.cn; 2 Beijing National Research Center for Information Science and Technology, Tsinghua University, Beijing 100084, China; 3 Aerospace Information Research Institute, Chinese Academy of Sciences, Beijing 100094, China; wangchunzhe0517@outlook.com; 4 Institute of Remote Sensing and Digital Earth, Beijing 100094, China; xigirl_jqw@163.com

**Keywords:** positioning error, wireless localization, tensor completion, sensor network, geometric dilution of precision (GDOP)

## Abstract

Accurate mapping of localization error distribution is essential for assessing passive sensor systems and guiding sensor placement. However, conventional analytical methods like the Geometrical Dilution of Precision (GDOP) rely on idealized error models, failing to capture the complex, heterogeneous error distributions typical of real-world environments. To overcome this challenge, we propose a novel data-driven framework that reconstructs high-fidelity localization error maps from sparse observations in TDOA-based systems. Specifically, we model the error distribution as a tensor and formulate the reconstruction as a tensor completion problem. A key innovation is our physics-informed regularization strategy, which incorporates prior knowledge from the analytical error covariance matrix into the tensor factorization process. This allows for robust recovery of the complete error map even from highly incomplete data. Experiments on a real-world dataset validate the superiority of our approach, showing an accuracy improvement of at least 27.96% over state-of-the-art methods.

## 1. Introduction

Passive localization is ubiquitous in fields ranging from wireless sensor networks (WSNs) to autonomous driving [1,2,3]. The reliability of these systems hinges on a thorough understanding of their performance across the operational domain. Consequently, a high-fidelity spatial localization error map is an indispensable tool, providing crucial insights for system evaluation, operational planning, and strategic sensor deployment. In practice, however, acquiring a complete and dense error map is often infeasible due to cost and logistical constraints, typically yielding only sparse measurements at discrete locations. Therefore, robustly reconstructing the full error distribution from limited data remains a significant challenge in the field.

Currently, the Geometric Dilution of Precision (GDOP) is the most widely adopted metric for performance evaluation [4,5]. The underlying principle is that measurement errors (e.g., in Time-Difference-of-Arrival, TDOA) are amplified by the geometric arrangement of the sensors, where GDOP provides a scalar metric to quantify this amplification factor [6,7]. Typically, unknown model parameters, such as measurement error variances, are estimated statistically from limited emitters and then extrapolated to unobserved regions. To enhance model fidelity in complex environments, numerous extensions to the basic GDOP model have been proposed. These include Weighted GDOP (WGDOP) variants accounting for heterogeneous noise in satellite navigation [8,9,10], as well as adaptations for indoor spaces [11,12], urban canyons [13], UAV-assisted networks [1,14,15], and WSNs [5,16].

Despite these refinements, model-driven methods share a fundamental limitation: they are predicated on idealized assumptions and oversimplified error models. They often fail to capture the complex coupling of error sources in real-world environment ssuch as non-line-of-sight (NLOS) propagation, multipath fading, and clock drift, leading to an unavoidable mismatch between predicted and actual system performance.

To mitigate this model mismatch, data-driven paradigms have emerged as a promising alternative. By learning directly from measurement data, these methods capture fine-grained environmental effects and complex error structures without relying on rigid physical models. Existing data-driven strategies for spatial map reconstruction generally fall into three categories. The first includes classical spatial interpolation techniques, such as Kriging [17,18] and kernel-based methods [19,20,21,22], which estimate values at unobserved locations based on spatial correlation. The second category comprises low-rank matrix and tensor completion methods, including compressed sensing [23], singular value thresholding [24,25,26], and tensor decomposition algorithms [27,28,29]. For instance, Zhang et al. [28] utilized block term decomposition (BTD) to reconstruct electromagnetic maps, while Sun et al. [30] improved accuracy by combining local interpolation with nuclear norm minimization (NNM-T). The third category encompasses machine learning approaches, such as RadioUNet [31], autoencoders [32], and Vision Transformer (ViT)-based methods [33]. While powerful, the efficacy of these purely data-driven methods is critically contingent on the availability of dense and uniformly distributed data [34,35]. In practical scenarios with sparse measurements, their performance degrades sharply, often failing to converge to physically meaningful solutions.

To overcome the limitations of inaccurate physical models and insufficient observational data, this paper proposes a framework that integrates model-based insights with the flexibility of data-driven approaches. First, we model the three-dimensional (3-D) spatial distribution of localization error as a third-order tensor, termed Tensorized GDOP (TGDOP). This representation offers a powerful mathematical tool to describe complex spatial error distributions, capturing intrinsic multi-dimensional structures and inherent anisotropy [36] that scalar metrics like GDOP fail to express. Subsequently, we formulate the reconstruction of the complete error map as a tensor completion problem. To solve this ill-posed problem under sparse measurement conditions, we develop a physics-informed regularization strategy. Specifically, we incorporate prior knowledge from the analytical error covariance matrix directly into the tensor factorization process by imposing polynomial constraints on the factor matrices. This physics-based constraint guides the reconstruction, ensuring the solution adheres to the underlying geometric principles of localization. It effectively compensates for missing observations, dramatically enhancing reconstruction accuracy and robustness without relying on simplified environmental models. Furthermore, this approach is not scenario-dependent and holds potential for extension to complex multi-system fusion scenarios.

Notably, while the concept of physics-informed learning is prominent in Physics-Informed Neural Networks (PINNs) [37], our usage differs fundamentally. PINNs typically integrate partial differential equations (PDEs) into a loss function and require training on large datasets to learn latent physical laws. In contrast, the proposed approach embeds these laws directly into the tensor structure as constraints for decomposition. Consequently, our method operates in a single-shot manner, which is training-free and requires no historical data. Experimental results on a real-world dataset demonstrate that, even with only 1% of observation data available, the proposed framework improves reconstruction accuracy by at least 27.96% compared to state-of-the-art baselines.

The main contributions of this paper are summarized as follows:

- Tensor-Based Spatial Error Modeling (TGDOP): We propose a novel framework that models the spatial distribution of positioning errors as a third-order tensor. Unlike conventional scalar GDOP metrics, this tensor representation explicitly captures the anisotropic characteristics and complex coupling of error sources in real-world 3-D environments.
- Physics-Informed Sparse Reconstruction Algorithm: We develop a robust tensor completion algorithm tailored for extremely sparse observational data. By deriving spatial properties from the theoretical error covariance matrix, we introduce polynomial constraints to the factor matrices during tensor decomposition.
- Training-Free and Model-Robust Performance: The proposed method operates as a single-shot, data-driven approach that does not require historical training data or idealized channel assumptions, validated on both simulated and real-world datasets.

The remainder of this paper is organized as follows: Section 2 describes the problem formulation, and Section 3 provides the preliminaries. The definition and properties of TGDOP are detailed in Section 4, followed by the sparse reconstruction algorithm in Section 5. Section 6 presents simulation and real-data experiments, and conclusions are drawn in Section 7.

## 2. Problem Statement

Consider a 3-D space discretized into a grid of $N_{1}\times N_{2}\times N_{3}$ cells. The coordinate corresponding to a grid cell with index (i,j,k)∈Ω is denoted by $u_{ijk}$, where Ω represents the set of all such indices, with  cardinality $\left|\Omega \right|=N_{1}N_{2}N_{3}$. Let $\Omega_{e}$ be the set of indices for grid cells containing emitters.

**Figure 1:**
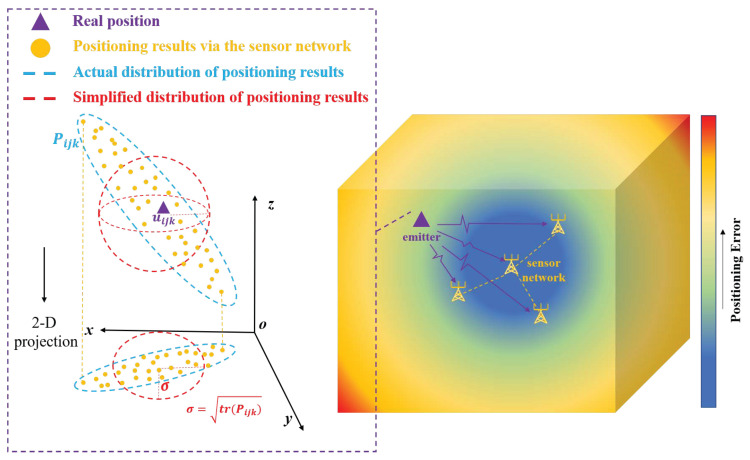
Schematicheatmap illustrating the magnitude of positioning error in a representative 3-D TDOA positioning system.

The characterization of positioning error begins with understanding its statistical nature at a specific location $u_{ijk}$. Assume there are *N* independent positioning results ${\hat{u}}_{ijk}^{n}$ (for n=1,…,N) for an emitter at a true location $u_{ijk}$, then the positioning error for each result is given by ${\mathrm{du}}_{ijk}^{n}={\hat{u}}_{ijk}^{n}-u_{ijk}$. The covariance matrix of the positioning error at $u_{ijk}$ is then calculated by

(1) $${\hat{P}}_{ijk}=E\left[{\mathrm{du}}_{ijk}{\mathrm{du}}_{ijk}^{T}\right],$$

where ${\mathrm{du}}_{ijk}=\left[\begin{array}{ccc} {\mathrm{du}}_{ijk}^{1} & \cdots & {\mathrm{du}}_{ijk}^{N} \end{array}\right]$. Suppose the positioning error measurements are available for any grid cell, then a heatmap of the spatial distribution of localization error can be generated by calculating the Root Mean Square Error (RMSE) value at each grid point, $\mathrm{RMSE}\left(u_{ijk}\right)=\sqrt{\mathrm{tr}({\hat{P}}_{ijk})}$, as  depicted in Figure 1.

In real-world applications, however, the acquisition of valid measurements is restricted to a very limited number of locations equipped with emitters. This inherent limitation means that statistical approaches can only characterize the positioning error locally and are incapable of perceiving the error map across the entire space. To overcome this, conventional methods use a parameterized empirical model for $P_{ijk}$ instead of a statistical one in (1), allowing extrapolation to emitter-free locations. These models typically define a few unknown parameters based on key error sources, which are then solved for using the limited available measurements. A typical example is the error map derived by the GDOP model for TDOA systems, which primarily accounts for errors in TDOA measurement noise $N(0,\sigma_{\Delta_{t_{l}}}^{2})$ and sensor position uncertainties $N(0,\sigma_{s}^{2})$:

(2) $$\begin{array}{cc} \; & \mathrm{GDOP}\left(u_{ijk}\right)=\sqrt{\mathrm{tr}(P_{ijk})},\;\mathrm{where}\\ \; & P_{ijk}=C_{ijk}\tilde{P}\left(\left\{\sigma_{\Delta_{t_{l}}}^{2}\right\},\sigma_{s}^{2}\right)C_{ijk}^{T}. \end{array}$$

Here, the specific forms of the geometry matrix $C_{ijk}$ and the intermediate parameter error covariance $\tilde{P}$ are detailed in Section 4.2. The variance of the *l*-th TDOA measurement, $\sigma_{\Delta_{t_{l}}}^{2}$, can be further expressed based on signal parameters [38] as

(3) $$\begin{array}{cc} \sigma_{\Delta_{t_{l}}} & =\frac{1}{\beta }\frac{1}{{BT\mathrm{SNR}}_{l}}, \end{array}$$

where β is the root mean square (RMS) signal bandwidth, *B* is the signal bandwidth, *T* is the RMS integration time, and ${\mathrm{SNR}}_{l}$ is the effective signal-to-noise ratio for the *l*-th TDOA calculation.

The parametric models can align with statistical estimates calculated by (1) if the underlying model assumptions are accurate. However, real-world scenarios involve complex propagation channels and diverse, often unmodeled, error sources. For instance, in typical NLOS scenarios, while the factors detailed (such as signal bandwidth and SNR) in (3) influence the standard deviation of TDOA measurements, the error component arising from NLOS propagation often plays a more dominant and decisive role. However, the GDOP model would be highly inaccurate because it does not account for NLOS errors. Moreover, the  need to design distinct models for $P_{ijk}$ tailored to different localization systems and specific application scenarios significantly curtails the practical utility and generalizability of conventional models.

To address the limitations of conventional models, the tensor-based framework surpasses conventional models by learning complex spatial distributions directly from data, independent of empirical models. We define the tensor representation of the 3-D positioning error map as $\underline{G}\in R^{N_{1}\times N_{2}\times N_{3}}$. Its components along the Cartesian axes (x, y, and z) are denoted as ${\underline{G}}_{1}$, ${\underline{G}}_{2}$, and ${\underline{G}}_{3}$, respectively. Then, the relationship between $\underline{G}$ and its components is given by

(4) $$\underline{G}=\sum_{r=1}^{3}{\underline{G}}_{r}.$$

In practical scenarios with spatially sparse emitters, the error statistics can only be measured at indices $\left(i,j,k\right)\in \Omega_{e}$. The measurement model for an element at index (i,j,k) is

(5) $$\begin{array}{cc} \hat{\underline{G}}\left(i,j,k\right) & =\mathrm{tr}\left({\hat{P}}_{ijk}\right)+\underline{N}\left(i,j,k\right),\\ {\hat{\underline{G}}}_{r}\left(i,j,k\right) & ={\hat{P}}_{ijk}\left(r,r\right)+{\underline{N}}_{r}\left(i,j,k\right),\;\left(r\in \left\{1,2,3\right\}\right), \end{array}$$

where $\underline{N}\left(i,j,k\right)$ and ${\underline{N}}_{r}\left(i,j,k\right)$ represent the observation errors associated with $\underline{G}$ and ${\underline{G}}_{r}$, respectively.

The learnable parameters in traditional models are limited to a predefined set of error sources from a prior model. In contrast, the tensor model’s parameters consist of the factor matrices and the core tensor (detailed in Section 4.1), where the factor matrices characterize the axial trends of the error distribution, and the core tensor represents the coupling relationships among these axial components. Thus, the tensor model can better adapt to complex real-world environments for inference in regions with no observations.

In this context, our primary problem is to reconstruct the complete underlying error component tensors ${\underline{G}}_{r}$ (and, consequently, the total error tensor $\underline{G}$) across the entire spatial domain Ω from sparse and noisy measurements ${\hat{\underline{G}}}_{r}\left(i,j,k\right)$ (and $\hat{\underline{G}}\left(i,j,k\right)$) available only at indices $\left(i,j,k\right)\in \Omega_{e}$. It is typically the case that the number of observed points is much smaller than the total number of grid points, i.e.,  $|\Omega_{e}\left|\ll |\Omega \right|$, and the observed locations $\Omega_{e}$ are often randomly distributed.

Notation: In this paper, we follow the established convention in signal processing. We denote scalars, vectors, matrices, and tensors with lowercase letters (a), boldface lowercase (a), boldface capitals (A), and underlined boldface capitals $\left(\underline{A}\right)$, respectively. $\underline{A}\left(i,j,k\right)$ denotes the (i,j,k)-th element of a third-order tensor. The colon notation indicates the sub-tensors of a given tensor. Italic capitals are also used to denote the index upper bounds. We use the superscripts $A^{-1}$ and $A^{†}$ to represent the inverse and the pseudo-inverse of a matrix, respectively. In addition, we use $∥\underline{A}{∥}_{F}=\sqrt{\sum_{i,j,k}a_{ijk}^{2}}$ to denote the Frobenius norms of a tensor. The operator diag(·) stacks its scalar arguments in a square diagonal matrix, cov(a,b) is the covariance of a and b, and tr(·) computes the trace of a matrix. The blockdiag(·) operator is defined as $\mathrm{blockdiag}\left(A_{1},\cdots ,A_{R}\right)=\left[\begin{array}{ccc} A_{1} & \cdots & 0\\ \vdots & \ddots & \vdots \\ 0 & \cdots & A_{R} \end{array}\right]$. The outer product follows that $a\circ b=\left[\begin{array}{c} ab_{j} \end{array}\right]$. The Hadamard product follows that $A⊙B=\left[\begin{array}{c} a_{ij}b_{ij} \end{array}\right]$. The Kronecker product is derived by $A⊗B=\left[\begin{array}{c} a_{ij}B \end{array}\right]$, and its capital symbol ${⨂}_{i}A_{i}$ is used to denote the multiplicative form. Then, the Khatri–Rao product is defined as the block-wise Kronecker product: $A{⊗}_{b}B=\left[\begin{array}{ccc} A_{1}⊗B_{1} & \cdots & A_{R}⊗B_{R} \end{array}\right]$. The $\delta_{ij}$ denotes the Kronecker delta. The (N×N) identity matrix is represented by $I_{N\times N}$, (M×N) zero matrix is denoted by $0_{M\times N}$, and $1_{N}$ is a column vector of all ones of length *N*.

## 3. Preliminaries

We begin with fundamental tensor analysis concepts to grasp the approach outlined in this paper. The issue discussed in this paper aligns with BTD, so the emphasis is on introducing the key concepts of BTD.

### 3.1. Mode-N Unfolding and Mode Product

For an *N*-th order tensor $\underline{X}\in R^{I_{1}\times I_{2}\times \cdots \times I_{N}}$, the mode-n unfolding of $\underline{X}$, denoted as ${\underline{X}}_{\left(n\right)}$, unfolds the *n*-th fiber as the columns of the resulting matrix. It maps the tensor element $x=\underline{X}\left(i_{1},i_{2},\cdots ,i_{N}\right)$ to the matrix element $\left(i_{n},j\right)$, where

(6) $$\begin{array}{cc} j= & 1+\sum_{k=1,k\neq n}^{N}\left(i_{k}-1\right)J_{k},\;with\;J_{k}=\prod_{l=1,l\neq n}^{k-1}I_{l}. \end{array}$$

The mode-n product of the $\underline{X}\in R^{I_{1}\times I_{2}\times \cdots \times I_{N}}$ with the $U\in R^{J\times I_{n}}$ is denoted as $\underline{X}\times_{n}U$, and it follows that

(7) $$\begin{array}{c} {\left(\underline{X}\times_{n}U\right)}_{i_{1}\cdots i_{n-1}ji_{n+1}\cdots i_{N}}=\sum_{i_{n}=1}^{I_{n}}x_{i_{1}i_{2}\cdots i_{N}}u_{ji_{n}}. \end{array}$$

Its matrix representation is expressed as

(8) $$\begin{array}{cc} \; & \underline{Y}=\underline{X}\times_{n}U,\;\mathrm{with}\;{\underline{Y}}_{\left(n\right)}=U{\underline{X}}_{\left(n\right)}. \end{array}$$

### 3.2. Block Tensor Decomposition in Multilinear Rank-(L,M,N) Terms

Unlike matrices, tensor rank determination is an NP-hard problem, leading to varied rank definitions among algorithms. The concept of multilinear rank is proposed in BTD. The tensor’s mode-n rank is defined as

**Definition** **1** (Mode-n rank)**.** *The mode-n rank of a tensor $\underline{X}$ is the dimension of the subspace spanned by its mode-n vectors.*

Then, a third-order tensor’s multilinear rank is rank−(L,M,N) if its mode-1 rank, mode-2 rank, and mode-3 rank are equal to *L*, *M*, and *N*, respectively.

A decomposition of a tensor $\underline{X}\in R^{I\times J\times K}$ in a sum of rank-(L,M,N) terms can be written as

(9) $$\underline{X}=\sum_{r=1}^{R}{\underline{S}}_{r}\times_{1}U_{r}\times_{2}V_{r}\times_{3}W_{r},$$

where ${\underline{S}}_{r}\in R^{L\times M\times N}$ is rank-(L,M,N), and $U_{r}\in R^{I\times L}$, $V_{r}\in R^{J\times M}$, and  $W_{r}\in R^{K\times N}$ are matrices with full column rank. Typically, the symbol ${\underline{S}}_{r}$ is referred to as the core tensor, while the matrices $U_{r}$, $V_{r}$, and $W_{r}$ are denoted as the factor matrices. Calculating the mode-n unfolding of (9), we have

(10) $$\begin{array}{cc} {\underline{X}}_{\left(1\right)}= & U\mathrm{blockdiag}\left({\left({\underline{S}}_{1}\right)}_{\left(1\right)},\cdots ,{\left({\underline{S}}_{R}\right)}_{\left(1\right)}\right){\left(W⊙V\right)}^{T},\\ {\underline{X}}_{\left(2\right)}= & V\mathrm{blockdiag}\left({\left({\underline{S}}_{1}\right)}_{\left(2\right)},\cdots ,{\left({\underline{S}}_{R}\right)}_{\left(2\right)}\right){\left(W⊙U\right)}^{T},\\ {\underline{X}}_{\left(3\right)}= & W\mathrm{blockdiag}\left({\left({\underline{S}}_{1}\right)}_{\left(3\right)},\cdots ,{\left({\underline{S}}_{R}\right)}_{\left(3\right)}\right){\left(V⊙U\right)}^{T}, \end{array}$$

where $U=\left[\begin{array}{ccc} U_{1} & \cdots & U_{R} \end{array}\right]$, $V=\left[\begin{array}{ccc} V_{1} & \cdots & V_{R} \end{array}\right]$, and  $W=\left[\begin{array}{ccc} W_{1} & \cdots & W_{R} \end{array}\right]$.

## 4. TGDOP and Its Properties

In this section, we introduce the TGDOP to characterize the distribution of localization errors and investigate the potential properties of TGDOP by analyzing the covariance matrix of positioning errors. Initially, we present a decomposed formulation of TGDOP, which explicitly accounts for the anisotropic characteristics inherent in localization errors. Subsequently, we analyze the general expressions for the error covariance matrix under various localization schemes. This analysis aims to establish the universality of the TGDOP model across different localization methodologies. Finally, by mapping the properties of the covariance matrix to the fundamental characteristics of the factor matrices within the TGDOP tensor space, we discuss the theoretical underpinnings that enable data-driven reconstruction using this model.

### 4.1. Tensor Model of Positioning Error Distribution

In real-world applications, conventional positioning error models are disturbed by model mismatch because their constrained parametric form limits their ability to capture the multiplicity of error sources found in complex environments. In contrast, tensor models are structured with far greater expressive power, allowing them to be effectively data-driven in constructing models that are sufficiently accurate and adaptable for practical applications.

The expressive power of tensor models stems from their core tensor and factor matrices. Analogous to the singular value decomposition (SVD) in 2-D cases, the 3-D tensor ${\underline{G}}_{r}$(r∈{1,2,3}) could be characterized by eigenspaces defined by three sets of eigenvectors. We denote these eigenspaces as $V_{1r}\in R^{N_{1}\times M_{1}}$, $V_{2r}\in R^{N_{2}\times M_{2}}$, and $V_{3r}\in R^{N_{3}\times M_{3}}$, corresponding to the factor matrices on the x, y, and z dimensions, respectively. Consequently, we have

(11) $$\begin{array}{cc} {\underline{G}}_{r}= & {\underline{S}}_{r}\times_{1}V_{1r}\times_{2}V_{2r}\times_{3}V_{3r}\;\left(r\in \left\{1,2,3\right\}\right), \end{array}$$

where ${\underline{S}}_{r}\in R^{M_{1}\times M_{2}\times M_{3}}$ is the core tensor of ${\underline{G}}_{r}$. Substituting (11) into (4), $\underline{G}$ can be expressed as

(12) $$\begin{array}{cc} \underline{G}= & \sum_{r=1}^{3}{\underline{S}}_{r}\times_{1}V_{1r}\times_{2}V_{2r}\times_{3}V_{3r}. \end{array}$$

The theoretical TGDOP model is presented in (12). Employing a BTD framework, this model effectively maps the spatial distribution of the diagonal components of the covariance matrix P onto a set of latent core tensors, ${\underline{S}}_{r}$. When this formulation is integrated with the expression for TGDOP measurements given in (5), it yields $\hat{\underline{G}}=\underline{G}+\underline{N}$. The probability distribution of the measurement noise $\underline{N}$ here satisfies Theorem 1.

**Theorem** **1** (Measurement Noise Distribution)**.** *Consider a 3-D scenario where an emitter is located at u, and there are N positioning results for this emitter. Let P represent the covariance matrix of the positioning error. When N is relatively larger than the diagonal elements in P, the observation noise of $\underline{\hat{G}}$ and ${\underline{\hat{G}}}_{r}$ at u follows that*

$$\begin{array}{cc} {\underline{N}}_{r}\left(u\right)\; & ∼N\left(0,\frac{2}{N}P^{2}\left(r,r\right)\right)\;\left(r\in \left\{1,2,3\right\}\right),\\ \underline{N}\left(u\right)\; & ∼N(0,\frac{2}{N}\sum_{r=1}^{3}P^{2}\left(r,r\right)). \end{array}$$

The proof is relegated to Appendix A. Theorem 1 establishes the condition under which measurements conform to a Gaussian distribution.

Notably, the TGDOP model presented in (12) involves not only the tensor $\underline{G}$ but also all its constituent core tensors and factor matrices. These elements enable the reconstruction of both $\underline{G}$ and its components, ${\underline{G}}_{r}$. In essence, TGDOP can be represented by a higher-order tensor $\underline{X}$ satisfying $\underline{X}\left(:,:,:,r\right)={\underline{G}}_{r}$. We demonstrate that $\underline{G}$, which lacks directional information, is a collapse of the directionally informed $\underline{X}$ along different dimensions. The relationship between $\underline{G}$ and $\underline{X}$ is further elucidated in Appendix C through the definition of a tensor operation.

### 4.2. The Covariance Matrix of Positioning Error

The covariance matrix of the positioning error is directly related to the TGDOP measurements, so the properties of the covariance matrix are the theoretical basis for designing the TGDOP factor matrix. Many documents have derived the covariance matrices of positioning errors under different positioning systems [36,39,40,41,42] in 2-D cases. This subsection proposes the covariance matrices of positioning errors for the TDOA positioning system in a 3-D Cartesian coordinate system.

Now, by defining the covariance matrix of the positioning error as $P\in R^{I\times J}$, we have I=J=3 for a 3-D scenario. Assume there are N sensors for TDOA positioning, where the main sensor and the auxiliary sensors are located at $u_{0}={\left[x_{0},y_{0},z_{0}\right]}^{T}$ and $u_{i}={\left[x_{i},y_{i},z_{i}\right]}^{T}\;\left(i=1,2,\cdots ,N-1\right)$, respectively, and the radiation source is located at $u={\left[x,y,z\right]}^{T}$. Let $\Delta t_{i}$ denote the TDOA measurements for the *i*-th group of sensors, $\sigma_{\Delta t_{i}}^{2}$ denote the variance of $\Delta t_{i}$, and $\sigma_{s}^{2}$ denote the variance of the sensor position error. The *i*-th row of the direction cosine matrix is given by

(13) $$\begin{array}{cc} F(i,:)= & \partial (∥u-u_{i}{∥}_{2}-∥u-u_{0}{∥}_{2})/\partial u\\ = & \left[\begin{array}{ccc} \frac{x-x_{i}}{r_{i,xyz}}-\frac{x-x_{0}}{r_{0,xyz}} & \frac{y-y_{i}}{r_{i,xyz}}-\frac{y-y_{0}}{r_{0,xyz}} & \frac{z-z_{i}}{r_{i,xyz}}-\frac{z-z_{0}}{r_{0,xyz}} \end{array}\right],\\ r_{i,xyz}= & \sqrt{{\left(x-x_{i}\right)}^{2}+{\left(y-y_{i}\right)}^{2}+{\left(z-z_{i}\right)}^{2}}. \end{array}$$

Let *c* denote the light speed, then the covariance matrix of the positioning error follows that $P=C\left(P_{\Theta }+P_{s}\right)C^{T}$, where

(14) $$\begin{array}{cc} C= & {\left(F^{T}F\right)}^{-1}F^{T},\\ P_{\Theta }\left(i,j\right)= & \left\{\begin{array}{ccc} \; & c^{2}\sigma_{\Delta t_{i}}^{2}\; & i=j\\ \; & c^{2}\eta_{ij}\sigma_{\Delta t_{i}}\sigma_{\Delta t_{j}}\; & i\neq j, \end{array}\right.\\ P_{s}\left(i,j\right)= & \left\{\begin{array}{ccc} \; & 2\sigma_{s}^{2}\; & i=j\\ \; & \sigma_{s}^{2}\; & i\neq j. \end{array}\right. \end{array}$$

Next, by defining $\tilde{P}=P_{\Theta }+P_{s}$ to represent the sum of the covariance matrices of all possible estimation errors, we have

(15) $$P=C\tilde{P}C^{T}.$$

It is found that under different localization regimes, the error covariance matrix P can consistently be derived from $\tilde{P}$ and C in a form analogous to (15). This is because (15) elucidates the principal sources of localization error. Specifically, $\tilde{P}$ represents the inherent estimation errors within the localization system, while C quantifies the anisotropic amplification effect on these errors attributable to the station geometry. Therefore, (15) can be considered a general expression for the localization error covariance matrix. Moreover, according to (5), the TGDOP measurements also incorporate this universal expressive capability, which explains its applicability across various localization systems.

### 4.3. Properties of TGDOP Derived from Error Covariance Matrix

A thorough understanding of its factor matrix properties is paramount to enable the effective reconstruction of the TGDOP model introduced in (12). This subsection investigates these properties by first examining the positioning error covariance matrix. We then translate the characteristics of this covariance matrix to the factor matrices in the tensor space, thereby establishing the smoothness, non-negativity, and low-rank properties of the proposed TGDOP model.

#### 4.3.1. Spatial Smoothness

The mathematical notation $C^{k}$ is commonly used to describe the smoothness of a function. Specifically, $C^{0}$ indicates that a function is continuous over its domain, while $C^{1}$ signifies that its first derivative exists and is also continuous over its domain (i.e., the function is continuously differentiable). Theorem 2 outlines the conditions for spatial continuity when the covariance matrix is treated as a matrix function of spatial position. A detailed derivation is available in Appendix B.

**Theorem** **2** (Continuity of Derivatives)**.** *$P\left(x,y,z\right)\in C^{1}\left(I\right)$ when $F:I\to R^{m\times n}$ satisfies the following conditions:*

- *$F\in C^{1}\left(I\right)$;*
- *∀(x,y,z)∈I,rank(F(x,y,z))=n.*

*(x, y, z) represent the position variables of the radiation source in the Cartesian coordinate system.*

Theorem 2 illustrates that the spatial continuity of F(x,y,z) plays a crucial role in the TGDOP model. Subsequently, we will analyze the continuity conditions for F(x,y,z) in the TDOA localization system. Calculate the $\nabla_{x}F\left(i,:\right)$:

(16) $$\begin{array}{cc} \nabla_{x}F\left(i,1\right)= & r_{i,xyz}^{-1}-r_{0,xyz}^{-1}+\frac{{\left(x-x_{0}\right)}^{2}}{r_{0,xyz}^{3}}-\frac{{\left(x-x_{i}\right)}^{2}}{r_{i,xyz}^{3}},\\ \nabla_{x}F\left(i,2\right)= & \frac{\left(x-x_{0}\right)\left(y-y_{0}\right)}{r_{0,xyz}^{3}}-\frac{\left(x-x_{i}\right)\left(y-y_{i}\right)}{r_{i,xyz}^{3}},\\ \nabla_{x}F\left(i,3\right)= & \frac{\left(x-x_{0}\right)\left(z-z_{0}\right)}{r_{0,xyz}^{3}}-\frac{\left(x-x_{i}\right)\left(z-z_{i}\right)}{r_{i,xyz}^{3}}, \end{array}$$

where $r_{i,xyz}$ is defined in (13). It can be readily observed that for an interval *I* where the condition $r_{i,xyz}\neq 0$ holds, the partial derivative $\nabla_{x}F$ is continuous on *I* (i.e., $\nabla_{x}F\in C^{0}\left(I\right)$). The forms of $\nabla_{y}F$ and $\nabla_{z}F$ are analogous to that of $\nabla_{x}F$, and thus, they adhere to the same continuity properties. Furthermore, F itself is continuous on *I* (i.e., $F\in C^{0}\left(I\right)$). Additionally, under a typical/standard sensor configuration, F possesses full column rank. Consequently, it can be readily concluded that $P\left(x,y,z\right)\in C^{1}\left(I\right)$ is based on Theorem 2.

According to (5) and (12), the property that $P\left(x,y,z\right)\in C^{1}\left(I\right)$ can be mapped to the tensor space. This implies that the dimension of the factor matrices’ solution space in (12) can be reduced through structured constraints, which is important for subsequent sparse reconstruction algorithms.

#### 4.3.2. Non-Negative

Theorem 3 states that the covariance matrix P is symmetric and positive semi-definite. Let $t={\left[1\;0\;0\right]}^{T}$. Then, for all $u_{ijk}\in \Omega$, we have

(17) $$t^{T}P_{ijk}t=P_{ijk}\left(1,1\right)\geq 0.$$

Considering the definition of ${\underline{G}}_{r}$ for r∈{1,2,3} in (5), it follows that ${\underline{G}}_{1}\geq 0$. By defining $t={\left[0\;1\;0\right]}^{T}$ and $t={\left[0\;0\;1\right]}^{T}$, respectively, similar conclusions can be drawn for ${\underline{G}}_{2}\geq 0$ and ${\underline{G}}_{3}\geq 0$. Leveraging this property, non-negative constraints are applied to the factor matrices of (12) in the subsequent algorithm. This confines the solution space to the domain of non-negative real numbers, reducing the variable dimensions in parameter optimization.

**Theorem** **3** (Symmetric Positive Semi-definite)**.** *Let $P=E[\mathrm{du}{\mathrm{du}}^{T}]$, where du denotes the positioning error vector. Then, P satisfies the following:*

- *$P^{T}=P$;*
- *$\forall t\in R^{n}\setminus \left\{0\right\},t^{T}Pt\geq 0$.*

**Proof.**  $$\begin{array}{cc} P^{T}= & E[{\left(\mathrm{du}{\mathrm{du}}^{T}\right)}^{T}]=P\\ t^{T}Pt= & t^{T}E\left[\mathrm{du}{\mathrm{du}}^{T}\right]t=E\left[t^{T}\mathrm{du}{\mathrm{du}}^{T}t\right]=E\left[{\left(t^{T}\mathrm{du}\right)}^{2}\right]\geq 0 \end{array}$$

   □

#### 4.3.3. Low Rank

To investigate the hypothesized low-rank property of the proposed tensor model $\underline{G}$, which is constructed from individual covariance matrices P as per (5), our approach is to analyze the rank characteristics of its constituent slices using SVD. The methodology involves simulating a scenario with high variability in the underlying error parameters to assess if low-rank structures persist even under such complex conditions.

The positioning error covariance matrix P at a location $u_{ijk}$ is generally expressed from (15) as $P\left(u_{ijk}\right)=C\left(u_{ijk}\right)\tilde{P}C{\left(u_{ijk}\right)}^{T}$. Here, $C(u_{ijk})$ is a geometry-dependent transformation matrix, and $\tilde{P}$ is a symmetric, positive, semi-definite matrix encapsulating the statistics of underlying sensor measurements or intermediate error parameters, influenced by environmental factors Θ. Performing a Cholesky decomposition on $\tilde{P}$ yields $\tilde{P}=LL^{T}$, where L is a lower triangular matrix. Consequently, the covariance matrix can be written as

(18) $$\begin{array}{c} P\left(u_{ijk}\right)=\left(C\left(u_{ijk}\right)L\right){\left(C\left(u_{ijk}\right)L\right)}^{T}. \end{array}$$

The matrix $C(u_{ijk})$ is determined by the fixed station configuration. Thus, the variability and specific structure of the positioning error map across different environments are primarily determined by L.

Assuming there are $N_{\mathrm{var}}$ independent intermediate variables contributing to $\tilde{P}$ (e.g., if *N* is the number of variables per sensor and *M* is the number of sensors or total measurements, then $N_{\mathrm{var}}$ could be M×N), $\tilde{P}$ is an $N_{\mathrm{var}}\times N_{\mathrm{var}}$ matrix, and thus $L\in R^{N_{\mathrm{var}}\times N_{\mathrm{var}}}$. We populate the non-zero elements of L with random values to simulate a complex error environment lacking strong prior structural information. The tensor $\underline{G}$ is then constructed using these generated $P(u_{ijk})$ matrices. We then perform SVD on matrix slices of $\underline{G}$. For instance, if a $\underline{G}\left(:,:,k\right)$ results in a matrix $G_{k}\in R^{D_{1}\times D_{2}}$ (for k=1,⋯,K), the normalized cumulative energy is defined as $\zeta_{i,k}=\sum_{j=1}^{i}\lambda_{j,k}/\sum_{j=1}^{J}\lambda_{j,k}$, where $\lambda_{j,k}$ is the *j*-th singular value of $G_{k}$ in descending order, and $J=\min(D_{1},D_{2})$. Then, the average of cumulative energy for *K* slices is defined as $\zeta_{i}=\frac{1}{K}\sum_{k=1}^{K}\zeta_{i,k}$.

**Figure 2:**
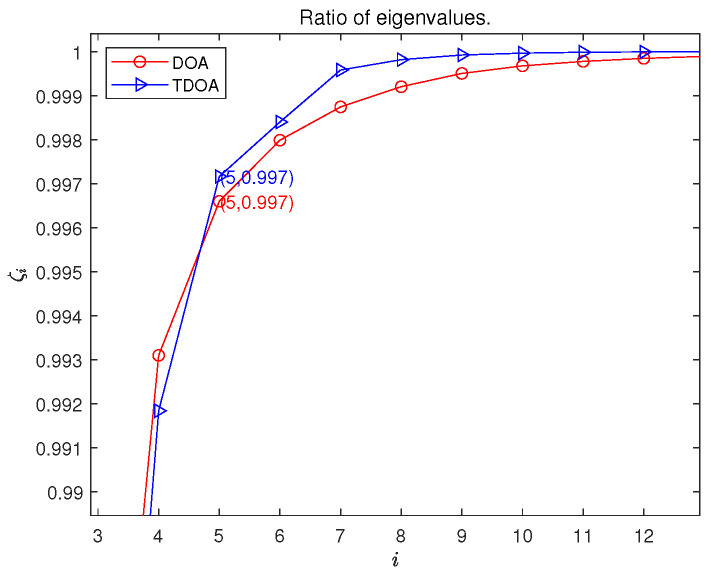
The plot of normalized cumulative singular value energy $\zeta_{i}$ versus singular value index *i* for the TGDOP, with L generated randomly. This indicates the potential low-rank property of the TGDOP.

Figure 2 plots $\zeta_{i}$ versus *i* for such an analysis, where the considered tensor slice dimensions result in J=80 singular values. It is found from Figure 2 that the first five singular values account for the majority of the energy in the analyzed tensor. This rapid decay of singular values suggests that the matrix representation of the positioning error distribution, even under these simulated complex conditions, exhibits a strong low-rank characteristic. This finding provides empirical support for the premise that the spatial distribution of positioning error can be effectively modeled by low-rank structures, offering a foundation for the low-rank reconstruction algorithm developed in this paper. While this analysis is based on a simulated scenario, the results suggest a general tendency that is further exploited by our proposed method.

## 5. Reconstruction Algorithm for TGDOP

In this section, we propose the sparse reconstruction algorithm for TGDOP. In cases where the positioning error sources are unidentified and limited emitters are available, we propose a BTD-based algorithm to enhance reconstruction performance under sparse measurements by constraining the factor matrix. In addition, we extend the algorithm when the directional measurements of positioning error are available. Specifically, we constrain the corresponding factor matrices with the directional measurements of positioning error in the objective function and reconstruct the optimized factor matrices into TGDOP.

### 5.1. BTD-Based Sparse Reconstruction Algorithm for TGDOP

We begin by outlining the context for the proposed algorithm. In this paper, a “sparse observational scenario” signifies a situation where calibration emitters are present in only a limited number of grids within the target area. Sensors receive electromagnetic signals from these emitters. Subsequent statistical analysis of multiple positioning results at each grid yields positioning errors; the squared magnitudes of these errors constitute the observational values, as detailed in (5).

As outlined in (12), the primary objective is the reconstruction of the complete positioning error tensor. This goal is achieved by solving the following optimization problem, which models the error tensor as a sum of *R* BTD components:

(19) $$\begin{array}{c} \min_{\begin{array}{c} \{{\underline{S}}_{r},V_{1r},\\ V_{2r},V_{3r}{\}}_{r=1}^{R} \end{array}}\frac{1}{2}{∥\underline{\hat{G}}-\sum_{r=1}^{R}\left({\underline{S}}_{r}\times_{1}V_{1r}\times_{2}V_{2r}\times_{3}V_{3r}\right)∥}_{F}^{2}. \end{array}$$

In this formulation, $\underline{\hat{G}}$ denotes the measurement tensor. Each term in the summation, ${\underline{S}}_{r}\times_{1}V_{1r}\times_{2}V_{2r}\times_{3}V_{3r}$, represents a BTD component comprising a core tensor ${\underline{S}}_{r}$ and associated factor matrices $V_{1r}\in R^{N_{1}\times M_{1}}$, $V_{2r}\in R^{N_{2}\times M_{2}}$, and $V_{3r}\in R^{N_{3}\times M_{3}}$. In accordance with (12), the number of BTD blocks is set to 3, representing the three directional components: x, y, and z. The dimensions $M_{k}$ satisfy $M_{k}\leq N_{k}$ (for k∈{1,2,3}), and these factor matrices are constrained to possess full column rank, a common requirement for model identifiability.

#### 5.1.1. Designing the Structure of Factor Matrices

Section 4.3 analyzes the intrinsic properties of the positioning error tensor $\underline{G}$. The factor matrices in the tensor decomposition represent projections of $\underline{G}$’s features along its different modes. Consequently, the  characteristics of $\underline{G}$ should inform the structure of these factor matrices. Theorem 2 reveals the spatial smoothness inherent in positioning errors. This implies that the column vectors of the factor matrices (which capture variations along spatial dimensions) can effectively represent most of the information using low-order polynomials. This prior knowledge is particularly valuable in scenarios with sparse observational data. Standard random initialization of factor matrices fails to incorporate this fundamental prior, potentially leading to models that are poorly constrained by sparse measurements and thus increasing the risk of convergence to suboptimal local minima.

Theorem 2 establishes the continuity of elements within the tensor of positioning error distribution. Here, we relate this property to the factor matrices of the tensor via Theorem 4, the detailed proof of which is provided in Appendix E.

**Theorem** **4** (Vandermonde-like structure)**.** *Given that the error distribution tensor G(x,y,z) exhibits $C^{1}$ continuity with respect to spatial position and possesses a low-rank structure, it follows that the column vectors of its factor matrices reside in a subspace generated by low-order polynomials.*

Based on Theorem 4, we propose structuring the column vectors of each factor matrix using polynomial generators. The optimization then focuses on two main steps. First, a  polynomial coefficient matrix is randomly initialized. Second, these coefficients are used to construct the columns of the actual factor matrix. Subsequent optimization iterations refine these polynomial coefficients rather than directly adjusting the elements of the factor matrices. This methodology explicitly incorporates the prior knowledge of spatial smoothness, promoting spatial continuity in the reconstructed tensor. Furthermore, by embedding this structural prior, the approach reduces the search space and guides the optimization, thereby decreasing the likelihood of converging to undesirable local optima.

Consider optimizing $V_{1r}$ in (19) as an illustrative example. We first randomly initialize a polynomial coefficient matrix $\Phi_{1r}\in R^{M_{1}\times m}$, where *m* is the number of coefficients used to define a polynomial of degree m−1. Each of the $M_{1}$ columns of $V_{1r}$ is generated using a distinct set of *m* polynomial coefficients. The initial factor matrix $V_{1r}^{\left(1\right)}$ is then constructed as

(20) $$\begin{array}{cc} V_{1r}^{\left(1\right)}= & \left[\begin{array}{cccc} 1 & 1 & \cdots & 1\\ 1 & 2 & \cdots & 2^{m-1}\\ \vdots & \vdots & \ddots & \vdots \\ 1 & N_{1} & \ldots & N_{1}^{m-1} \end{array}\right]\Phi_{1r}^{T}\\ = & Q\Phi_{1r}^{T}, \end{array}$$

where $Q\in R^{N_{1}\times m}$ is the Vandermonde matrix whose columns form a basis for polynomials up to degree m−1. Since Q is full column rank (given $m\leq N_{1}$), optimizing the objective function with respect to $V_{1r}^{\left(1\right)}$ is equivalent to optimizing with respect to $\Phi_{1r}$. The optimization task thus shifts from determining $V_{1r}$ (with $M_{1}N_{1}$ parameters) to determining $\Phi_{1r}$ (with $M_{1}m$ parameters), significantly reducing the dimension of parameters to be optimized in the solution space when $m\ll N_{1}$. This reduction effectively smooths the optimization landscape and prevents the algorithm from getting trapped in bad local minima associated with high-frequency noise, thereby improving the robustness to random initialization.

To ensure the non-negativity of the factor matrices indicated in Theorem 3, we define the final factor matrix $V_{1r}^{\left(2\right)}$ through an element-wise squaring operation:

(21) $$\begin{array}{c} V_{1r}^{\left(2\right)}=V_{1r}^{\left(1\right)}⊙V_{1r}^{\left(1\right)}. \end{array}$$

Upon substituting Equations (20) and (21) into the objective function (denoted as H) defined in (19), the optimization problem concerning the factor matrix $V_{1r}$ can be re-expressed as

(22) $$\begin{array}{c} \min_{V_{1r}^{\left(2\right)}}H\left(V_{1r}^{\left(2\right)}\right)=\min_{\Phi_{1r}}H\left(\left(Q\Phi_{1r}^{T}\right)⊙\left(Q\Phi_{1r}^{T}\right)\right). \end{array}$$

Consequently, optimizing the objective function H from (19) concerning the factor matrix $V_{1r}$ (now denoted $V_{1r}^{\left(2\right)}$ to reflect its construction) becomes equivalent to optimizing for the polynomial coefficient matrix $\Phi_{1r}$. For brevity, any subsequent reference to optimizing a factor matrix implies the optimization of its underlying polynomial parameter matrix Φ.

#### 5.1.2. Sparse Formulation of the Objective Function

In practical applications, tensor reconstruction often addresses scenarios with sparse measurements, where the measured data $\underline{\hat{G}}$ covers only a minor portion of the entire target area. To model this, we introduce a binary sampling tensor $\underline{W}$, where $\underline{W}\left(i,j,k\right)=1$ if the element (i,j,k) is observed (i.e., $\left(i,j,k\right)\in \Omega_{e}$), and 0 otherwise. Assuming the unobserved entries in $\underline{\hat{G}}$ are represented as zeros, the optimization problem for sparse data is formulated as

(23) $$\begin{array}{cc} \; & \min_{\begin{array}{c} {\underline{S}}_{r},V_{1r},\\ V_{2r},V_{3r} \end{array}}\frac{1}{2}{∥\underline{\hat{G}}-\tilde{\underline{G}}∥}_{F}^{2},\;\mathrm{with}\\ \; & \tilde{\underline{G}}=\sum_{r=1}^{R}\underline{W}⊙\left({\underline{S}}_{r}\times_{1}V_{1r}\times_{2}V_{2r}\times_{3}V_{3r}\right). \end{array}$$

#### 5.1.3. Solving Equation (23) Using Block Coordinate Descent

Now, we employ a Block Coordinate Descent (BCD) strategy to solve (23), which involves iteratively minimizing the objective function with respect to one block of variables while keeping the others fixed. This leads to the following four sub-problems, derived from the mode-n matricized forms of the objective function:

(24) $$\begin{array}{cc} \; & \min_{V_{1}}{∥{\underline{\hat{G}}}_{\left(1\right)}-{\underline{W}}_{\left(1\right)}⊙\left(V_{1}\mathrm{blockdiag}{\left({\underline{S}}_{1:R}\right)}_{\left(1\right)}{\left(V_{3}{⊗}_{b}V_{2}\right)}^{T}\right)∥}_{F}^{2},\\ \; & \min_{V_{2}}{∥{\underline{\hat{G}}}_{\left(2\right)}-{\underline{W}}_{\left(2\right)}⊙\left(V_{2}\mathrm{blockdiag}{\left({\underline{S}}_{1:R}\right)}_{\left(2\right)}{\left(V_{3}{⊗}_{b}V_{1}\right)}^{T}\right)∥}_{F}^{2},\\ \; & \min_{V_{3}}{∥{\underline{\hat{G}}}_{\left(3\right)}-{\underline{W}}_{\left(3\right)}⊙\left(V_{3}\mathrm{blockdiag}{\left({\underline{S}}_{1:R}\right)}_{\left(3\right)}{\left(V_{2}{⊗}_{b}V_{1}\right)}^{T}\right)∥}_{F}^{2},\\ \; & \min_{\left\{{\underline{S}}_{r}\right\}}{∥\mathrm{vec}\left(\underline{\hat{G}}\right)-\mathrm{vec}\left(\underline{W}\right)⊙\left(\left(V_{3}{⊗}_{b}V_{2}{⊗}_{b}V_{1}\right)\mathrm{vec}\left({\underline{S}}_{1:R}\right)\right)∥}_{F}^{2}, \end{array}$$

where

(25) $$\begin{array}{cc} \; & V_{k}=\left[V_{k1},\cdots ,V_{kR}\right],\;\mathrm{for}\;k=1,2,3,\\ \; & \mathrm{blockdiag}{\left({\underline{S}}_{1:R}\right)}_{\left(i\right)}=\mathrm{blockdiag}\left\{{\left({\underline{S}}_{1}\right)}_{\left(i\right)},\cdots ,{\left({\underline{S}}_{R}\right)}_{\left(i\right)}\right\},\\ \; & \mathrm{vec}\left({\underline{S}}_{1:R}\right)=\left[\mathrm{vec}\left({\underline{S}}_{1}\right);\cdots ;\mathrm{vec}\left({\underline{S}}_{R}\right)\right]. \end{array}$$

The variables to be optimized in (23) are the sets of factor matrices ${\left\{V_{1r}\right\}}_{r=1}^{R}$, ${\left\{V_{2r}\right\}}_{r=1}^{R}$, ${\left\{V_{3r}\right\}}_{r=1}^{R}$ (collectively denoted $V_{1},V_{2},V_{3}$), and the set of core tensors ${\left\{{\underline{S}}_{r}\right\}}_{r=1}^{R}$. Denoting the objective function in (23) by $F(V_{1},V_{2},V_{3},\left\{{\underline{S}}_{r}\right\})$, the  BCD method iteratively solves the following:

(26) $$\begin{array}{cc} V_{1}^{\left(t+1\right)} & =\underset{V_{1}}{\arg\;\min}F\left(V_{1},V_{2}^{\left(t\right)},V_{3}^{\left(t\right)},\left\{{\underline{S}}_{r}^{\left(t\right)}\right\}\right),\\ V_{2}^{\left(t+1\right)} & =\underset{V_{2}}{\arg\;\min}F\left(V_{1}^{\left(t+1\right)},V_{2},V_{3}^{\left(t\right)},\left\{{\underline{S}}_{r}^{\left(t\right)}\right\}\right),\\ V_{3}^{\left(t+1\right)} & =\underset{V_{3}}{\arg\;\min}F\left(V_{1}^{\left(t+1\right)},V_{2}^{\left(t+1\right)},V_{3},\left\{{\underline{S}}_{r}^{\left(t\right)}\right\}\right),\\ \left\{{\underline{S}}_{r}^{\left(t+1\right)}\right\} & =\underset{\left\{{\underline{S}}_{r}\right\}}{\arg\;\min}F\left(V_{1}^{\left(t+1\right)},V_{2}^{\left(t+1\right)},V_{3}^{\left(t+1\right)},\left\{{\underline{S}}_{r}\right\}\right). \end{array}$$

Each sub-problem in (26) is a linear least squares problem concerning the optimized variable block and is therefore convex. Consequently, an Alternating Least Squares (ALS) approach, a specific type of BCD, can be employed, as detailed in Algorithm 1.
**Algorithm 1** ALS algorithm for solving (23).Initialize $V_{1},V_{2},V_{3},{\left\{{\underline{S}}_{r}\right\}}_{r=1}^{R}$.**while** not converged **do**    **for** i=1,2,3 **do**        **Update** $V_{i}=\left[V_{i1},\ldots ,V_{iR}\right]$:        $B\leftarrow \mathrm{blockdiag}{\left({\underline{S}}_{1:R}\right)}_{\left(i\right)}{\left({⨂}_{j=3,j\neq i}^{1}V_{j}\right)}^{T}$.        **for** $j=1,\ldots ,N_{1}$ **do**         $w\leftarrow {\underline{W}}_{\left(i\right)}\left(j,:\right)$.         $V_{i}\left(j,:\right)\leftarrow \left(\left({\underline{\hat{G}}}_{\left(i\right)}\left(j,:\right)⊙w\right)B^{T}\right){\left(B\mathrm{diag}\left(w\right)B^{T}\right)}^{†}$.        **end for**        **for** r=1,…,R **do**         Perform QR-factorization: $V_{ir}=Q_{ir}R_{ir}$.         $V_{\mathrm{ir}}\leftarrow Q_{ir}$, ${\underline{S}}_{r}\leftarrow {\underline{S}}_{r}\times_{i}R_{ir}$.        **end for**    **end for**    **Update** ${\left\{{\underline{S}}_{r}\right\}}_{r=1}^{R}$:        $A\leftarrow V_{3}{⊗}_{b}V_{2}{⊗}_{b}V_{1}$, $w\leftarrow \mathrm{vec}(\underline{W})$.        $\mathrm{vec}\left({\underline{S}}_{1:R}\right)\leftarrow {\left(A^{T}\mathrm{diag}\left(w\right)A\right)}^{†}A^{T}\mathrm{diag}\left(w\right)\mathrm{vec}\left(\underline{\hat{G}}\right)$.**end while**Reconstruct $\underline{G}$ and ${\underline{G}}_{r}$ using converged factors via (11) and (12), respectively.**Return**$\;\underline{G}$ and ${\underline{G}}_{r}$.

**Figure 3:**
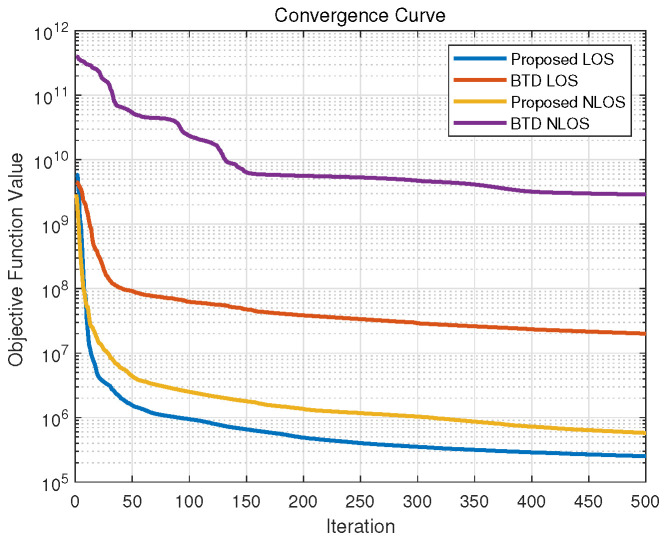
The average convergence curves of the proposed algorithm and the classical BTD algorithm in LOS and NLOS scenarios, with 100 random initializations.

The focus of this study lies in tensor modeling methodology, so we employed a straightforward ALS-BCD optimization scheme without an exhaustive comparison of alternative reconstruction strategies. Figure 3 illustrates the average convergence curves of the proposed algorithm and the classical BTD algorithm in LOS and NLOS scenarios, based on 100 random initializations. In the figure, the x-axis represents the number of optimization steps performed by the ALS algorithm, and the y-axis represents the total squared Frobenius norm error defined in (23). This value is calculated based on the normalized measurement tensor and is used to illustrate the relative magnitude of the convergence trend. It can be observed that the proposed algorithm outperforms the classical BTD method in terms of both convergence speed and stability, and exhibits sufficient robustness to initial value selection. Nevertheless, to extend the algorithm to larger tensor spaces, advanced techniques such as momentum-based updates, stochastic optimization, or distributed computation can be incorporated to further boost convergence speed and robustness.

For the aforementioned solution process, the derivation of its computational complexity is provided in Appendix D:

(27) $$C_{\mathrm{total}}\approx O\left(|\Omega |\;\cdot \;R^{2}{\left(M_{1}M_{2}M_{3}\right)}^{2}\right),$$

where |Ω| denotes the number of observed samples. It is evident that the complexity of the proposed algorithm is acceptable under the low-rank assumption. However, when the multilinear rank is large, reconstruction accuracy must be sacrificed to reduce time costs. Future research may overcome this technical bottleneck by modifying the optimization strategy.

### 5.2. Solution Approach with Available Directional Measurements

The rotational ambiguity of sub-tensors is a known challenge in tensor decomposition and is often handled with prior constraints, initialization, or post-processing [28]. This subsection explores an alternative solution strategy when directional components of the positioning error are available as measurements. It is emphasized that the proposed method does not decompose the tensor into three arbitrary blocks. Instead, it reconstructs three physically meaningful components grounded in the definition of the error covariance matrix. This ensures that the physical correspondence is guaranteed by the model’s design from the outset, rather than being an ambiguous interpretation of the decomposition results.

While existing research on positioning errors has largely concentrated on scalar distributions (e.g., GDOP-based models) that overlook directional characteristics, such information is not irretrievable. When a set of N positioning measurements ${\left\{u_{i}\right\}}_{i=1}^{N}$ is collected for an emitter at a known location u, the directional error components ${\underline{\hat{G}}}_{r}$ can be extracted through statistical analysis.

The availability of these directional measurements ${\underline{\hat{G}}}_{r}$ enables a refined modeling approach. We can reformulate the objective function to model each of the *R* observed directional tensors with a dedicated BTD component, leading to the expression:

(28) $$\begin{array}{cc} \min_{\begin{array}{c} {\underline{S}}_{r},V_{1r},\\ V_{2r},V_{3r} \end{array}}\; & \frac{1}{2}\sum_{r=1}^{R}{∥{\underline{\hat{G}}}_{r}-\underline{W}⊙\left({\underline{S}}_{r}\times_{1}V_{1r}\times_{2}V_{2r}\times_{3}V_{3r}\right)∥}_{F}^{2}. \end{array}$$

This objective would then be decomposed into sub-problems:

(29) $$\begin{array}{cc} \; & \min_{V_{1r}}\sum_{r}{∥{\underline{\hat{G}}}_{r(1)}-{\underline{W}}_{\left(1\right)}⊙\left(V_{1r}{\underline{S}}_{r(1)}{\left(V_{3r}⊗V_{2r}\right)}^{T}\right)∥}_{F}^{2},\\ \; & \min_{V_{2r}}\sum_{r}{∥{\underline{\hat{G}}}_{r(2)}-{\underline{W}}_{\left(2\right)}⊙\left(V_{2r}{\underline{S}}_{r(2)}{\left(V_{3r}⊗V_{1r}\right)}^{T}\right)∥}_{F}^{2},\\ \; & \min_{V_{3r}}\sum_{r}{∥{\underline{\hat{G}}}_{r(3)}-{\underline{W}}_{\left(3\right)}⊙\left(V_{3r}{\underline{S}}_{r(3)}{\left(V_{2r}⊗V_{1r}\right)}^{T}\right)∥}_{F}^{2},\\ \; & \min_{{\underline{S}}_{r}}\sum_{r}{∥\mathrm{vec}\left({\underline{\hat{G}}}_{r}\right)-\mathrm{vec}\left(\underline{W}\right)⊙\left(\left(V_{3r}⊗V_{2r}⊗V_{1r}\right)\mathrm{vec}\left({\underline{S}}_{r}\right)\right)∥}_{F}^{2}. \end{array}$$

Utilizing directional measurements aims to provide richer constraints for the factor matrices and core tensors, potentially improving the accuracy of directional positioning error reconstruction. Similar to Algorithm 1, a BCD approach can be applied to solve these convex sub-problems; the detailed algorithmic steps are omitted here for brevity.

### 5.3. Lower Bound on Sample Complexity

In this section, we discuss the lower bound of the data requirement of the proposed algorithm under the random uniform sampling strategy. We consider the recovery of a *K*-th order tensor $\underline{G}\in R^{N_{1}\times \cdots \times N_{K}}$, which is represented as a sum of *R* low-rank Tucker tensors ${\left\{T_{r}\right\}}_{r=1}^{R}$: $\underline{G}=\sum_{r=1}^{R}T_{r}$. Each component tensor $T_{r}$ has a Tucker decomposition:

(30) $$T_{r}=S_{r}\times_{1}V_{r,1}\times_{2}V_{r,2}\cdots \times_{K}V_{r,k},$$

where $S_{r}$ is the core tensor and $V_{r,k}\in R^{N_{k}\times d_{rk}}$ is the factor matrix for component *r* along mode *k*, with $d_{rk}$ being the mode-*k* Tucker rank. The analysis results on Tucker decomposition [43,44] indicate that the lower bound for the sample complexity is determined by the most difficult-to-recover matrix unfolding, $T_{\left(k\right)}$. The recovery difficulty of a matrix is lower-bounded by a function proportional to $\mathrm{rank}\times (\dim_{1}+\dim_{2})$, that is,

(31) $$z\geq \max_{\;k\in \{1,\ldots ,K\}}\left\{\Omega \left(\mathrm{rank}\left(T_{\left(k\right)}\right)\left(N_{k}+\prod_{j\neq k}N_{j}\right)\right)\right\}.$$

In Section 5.1.1, we assume that each column of every factor matrix $V_{r,k}$ is a polynomial sampled at $N_{k}$ distinct points. This structural prior can be formulated as a factorization $V_{r,k}=Q_{r,k}{\Phi_{r,k}}^{⊤}$, where $Q_{r,k}\in R^{N_{k}\times m_{r,k}}$ and $\Phi_{r,k}\in R^{d_{rk}\times m_{r,k}}$. Here, $d_{rk}$ represents the mode-k rank of the r-th component of the tensor, and $m_{r,k}$ represents the order used for polynomial approximation. Each row of $\Phi_{r,k}$ contains the coefficients for the corresponding column of $V_{r,k}$ in the basis defined by $Q_{r,k}$.

The mode-*k* unfolding of the *r*-th component tensor, ${\left(T_{r}\right)}_{\left(k\right)}$, is given by

(32) $${\left(T_{r}\right)}_{\left(k\right)}=V_{r,k}{\left(S_{r}\right)}_{\left(k\right)}{\left(V_{r,k}⊗\cdots ⊗V_{r,k+1}⊗V_{r,k-1}⊗\cdots ⊗V_{r,1}\right)}^{T}.$$

Substituting the polynomial constraint $V_{r,j}=Q_{r,j}\Phi_{r,j}^{⊤}$ for all modes j=1,…,K, we obtain

(33) $${\left(T_{r}\right)}_{\left(k\right)}=\left(Q_{r,k}\Phi_{r,j}^{⊤}\right){\left(S_{r}\right)}_{\left(k\right)}{\left(\underset{j\neq k}{⨂}\left(Q_{r,j}\Phi_{r,j}^{⊤}\right)\right)}^{T}.$$

The expression for ${\left(T_{r}\right)}_{\left(k\right)}$ shows that its column space is a subspace of the column space of the known matrix $Q_{r,k}$:

(34) $$\mathrm{col}\left({\left(T_{r}\right)}_{\left(k\right)}\right)\subseteq \mathrm{col}\left(Q_{r,k}\right).$$

The search for the left singular vectors is thus restricted from the ambient $R^{N_{k}}$ space to the $m_{r,k}$-dimensional subspace spanned by the columns of $Q_{r,k}$. The effective row dimension is reduced from $N_{k}$ to $m_{r,k}$. Using the mixed-product property of the Kronecker product on the right-hand side of the expression:

(35) $$\underset{j\neq k}{⨂}\left(Q_{r,j}\Phi_{r,j}^{⊤}\right)=\left(\underset{j\neq k}{⨂}Q_{r,j}\right)\left(\underset{j\neq k}{⨂}\Phi_{r,j}^{⊤}\right).$$

This implies that the row space of ${\left(T_{r}\right)}_{\left(k\right)}$ is contained within the column space of the matrix ${⨂}_{j\neq k}Q_{r,j}$, whose dimension is $\prod_{j\neq k}m_{r,j}$. The effective column dimension is therefore reduced from $\prod_{j\neq k}N_{j}$ to $\prod_{j\neq k}m_{r,j}$.

For the matrix recovery problem of ${\left(T_{r}\right)}_{\left(k\right)}$, we conclude that it is rank-$d_{rk}$ with effective $m_{r,k}$ row dimension and $\prod_{j\neq k}m_{r,j}$ column dimension. Applying the standard lower bound for matrix recovery yields the bound for this specific unfolding:

(36) $$L_{r}^{\left(k\right)}=\Omega \left(d_{rk}\left(m_{r,k}+\prod_{j\neq k}m_{r,j}\right)\right).$$

Theorem 5 gives the overall sample complexity lower bound for the BTD recovery problem, showing that the sample complexity is determined by the bottleneck among all R×K unfoldings.

**Theorem** **5.** *For the recovery of a BTD tensor with polynomial factor matrix constraints, the required number of measurements m has the following lower bound:*

(37) $$z\geq \max_{r\in \{1,\ldots ,R\},\;k\in \{1,\ldots ,K\}}\left\{\Omega \left(d_{rk}\left(m_{r,k}+\prod_{j\neq k}m_{r,j}\right)\right)\right\},$$

*where $d_{rk}$ is the Tucker rank and $m_{r,k}$ is the degree of freedom for the factor matrix of component r along mode k.*

**Figure 4:**
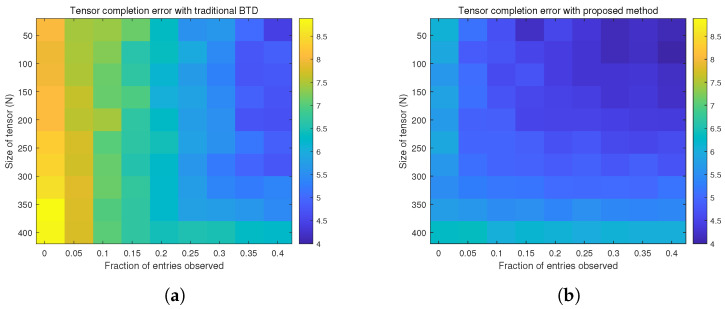
Analysis of the impact of sample size on the performance of tensor reconstruction. (**a**) Reconstruction error of the conventional BTD model. (**b**) Reconstruction error of the BTD model with factor matrix prior constraints.

Figure 4 shows the log-reconstruction error under different observation ratios. The x-axis represents a ratio between 0 and 1, indicating the proportion of observed data points relative to the total number of tensor elements. The y-axis denotes the size N of the N×N×N three-dimensional tensor. The colorbar of the heatmap represents the reconstruction error on a logarithmic scale. The performance of the conventional BTD algorithm and the proposed method is presented in Figure 4a and Figure 4b, respectively. The results indicate that under identical sampling conditions, our method exhibits superior performance by achieving a lower reconstruction error. This consequently reduces the sample lower bound for achieving high-fidelity tensor reconstruction.

## 6. Results with Measurements and Simulations

To evaluate the performance and robustness of the proposed algorithm under diverse conditions, we conduct simulations on two typical TDOA-based positioning scenarios, denoted as $S_{TDOA}$ and $S_{TDOA}^{N}$, respectively. The experimental findings and conclusions can also be extended to Direction-of-Arrival (DOA) positioning scenarios. Nevertheless, specific results from DOA experiments are not further detailed in this paper for brevity.

The scenario $S_{TDOA}$ utilizes a 3-D TDOA positioning system with four sensors. Three sets of TDOA measurements are the intermediate parameters. The standard deviations for these TDOA measurement errors are set to 18 ns, 20 ns, and 25 ns. The standard deviation of sensor position errors is modeled to be 0.5 m. The sensor coordinates (in meters) are ${\left[0,0,0\right]}^{T}$, ${\left[640,1070,-35\right]}^{T}$, ${\left[-900,-180,-27\right]}^{T}$, and ${\left[1000,-660,-35\right]}^{T}$, forming a “Y”-shaped constellation. This configuration is chosen not only because it represents the most common and high-performing geometry in distributed positioning, but also to align with the experimental setup described later. Due to a shared reference sensor in TDOA calculations, correlations are introduced among the TDOA measurement errors. Let the TDOA error vector be $\epsilon_{\mathrm{TDOA}}={\left[\epsilon_{1},\epsilon_{2},\epsilon_{3}\right]}^{T}$. The correlation matrix $R_{\epsilon }$ for these TDOA errors has $\rho_{12}=-0.3,\rho_{13}=0.5,\;\mathrm{and}\;\rho_{23}=-0.2$. The maximum simulated detection range extends to 8 km, with the target area’s elevation spanning from 0.5 km to 5 km. This target volume is discretized into a grid of 810×810×110 points. Scenario $S_{\mathrm{TDOA}}^{N}$ builds upon $S_{\mathrm{TDOA}}$ by incorporating multipath errors within a quarter of the region spanning elevations from 0.5 km to 1.4 km. The multipath error affecting the TDOA estimates is modeled using an exponential distribution with a mean of 100 ns.

To quantitatively assess the algorithm’s performance, several error metrics were employed. Mean Frobenius Norm Error (MFNE) is used to evaluate the average reconstruction error of a tensor $\underline{G}$ over multiple trials: ${\mathrm{MFNE}}_{\underline{G}}=\frac{1}{M}\sum_{m=1}^{M}∥\underline{G}-{\underline{\hat{G}}}_{m}{∥}_{F}$, where $\underline{G}$ is the true tensor, ${\underline{\hat{G}}}_{m}$ is the tensor reconstructed in the *m*-th Monte Carlo trial, and *M* is the total number of trials. Relative Frobenius Norm Error (RFNE) measures the reconstruction error relative to the magnitude of the true tensor: ${\mathrm{RFNE}}_{\underline{G}}=\frac{∥\underline{G}-{\underline{\hat{G}}}_{m}{∥}_{F}}{∥\underline{G}{∥}_{F}}$. This metric is typically calculated for each trial or averaged over *M* trials. Signal-to-Noise Ratio (SNR) represents the ratio of the true signal power to the noise power in the measurements, defined in decibels (dB) as ${\mathrm{SNR}}_{\underline{G}}=10\log_{10}\left(\frac{∥\underline{G}{∥}_{F}}{∥{\underline{G}}_{\mathrm{noisy}}-\underline{G}{∥}_{F}}\right)$, where $\underline{G}$ is the true underlying (noise-free) tensor and ${\underline{G}}_{\mathrm{noisy}}$ is the observed noisy tensor.

### 6.1. Multilinear Rank Analysis

In this subsection, we employ the L-curve method (Note: The L-curve corner identifies the inflection point where further increases in model parameters yield diminishing returns in error reduction; the rank at this corner is selected as the optimal multilinear rank to balance accuracy and model simplicity.) to determine a suitable multilinear rank for the tensor model optimized by our proposed algorithm. Given that the concept of low rank is relative to a tensor’s dimensions, we estimate the multilinear rank for the target tensor.

To manage computational load during rank analysis, especially for large tensors, we introduce a down-sampling factor $f_{s}$. The analysis is performed on a down-sampled version of the target data tensor, denoted as $\underline{G}\in R^{\left\lfloor N_{1}/f_{s}\right\rfloor \times \left\lfloor N_{2}/f_{s}\right\rfloor \times \left\lfloor N_{3}/f_{s}\right\rfloor }$. The L-curve method typically involves plotting a measure of reconstruction error against model complexity. The corner of this curve often indicates an optimal trade-off. We identify this region by conducting trials with various combinations of multilinear ranks and select the ranks near the L-curve’s inflection point. This selection is further guided by the criterion that the RFNE between the $\underline{G}$ and its low-rank approximation $\underline{\hat{G}}$ falls below a predefined threshold $2\times 10^{-3}$.

**Figure 5:**
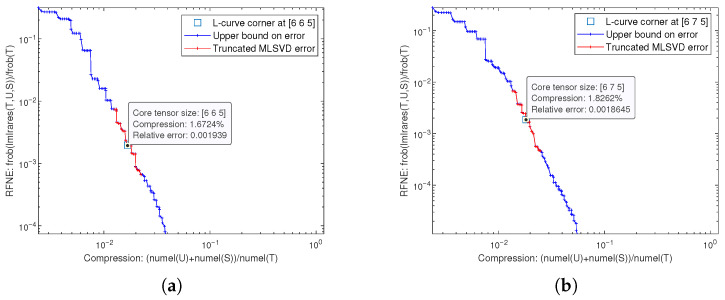
Multilinear rank estimation via the L-curve method at $f_{s}=20$. Iterate through multilinear ranks and select the first point in the Pareto front set with an error below the predefined threshold as the L-curve corner. (**a**) The L-curve for scenario $S_{\mathrm{TDOA}}$. (**b**) The L-curve for scenario $S_{\mathrm{TDOA}}^{N}$.

Figure 5 illustrates the estimation of the tensor’s multilinear rank using the L-curve method at $f_{s}=20$. The x-axis represents the compression ratio, defined as the percentage of parameters required for reconstruction relative to the total tensor size, while the y-axis denotes the RFNE. The selection of $f_{s}=20$ provides an optimal trade-off, because it reduces the number of elements by a factor of $f_{s}^{3}$, enabling rapid iterative rank searching while maintaining the macro-scale spatial correlation of the error distribution as guaranteed by the continuity of the TGDOP model. Furthermore, extra experimental results also reveal that across various down-sampling factors $f_{s}$ from 1 to 20, the estimated multilinear rank for each mode consistently remained below 10% of that mode’s original dimension, underscoring the inherent low-rank nature of the data.

### 6.2. Performance Under Sparse Measurements

This subsection evaluates the algorithm’s reconstruction performance when the emitters are sparsely and randomly distributed within the target area. We define the missing emitter ratio (MER) as the proportion of grid points in the target area lacking emitter data [45]. We conducted experiments with MER varying from 80% to 99% (corresponding to an observation ratio of 20% to 1%) to assess performance under increasing sparsity where the observation ratio is defined as 100% MER. For these experiments, the down-sampling factor $f_{s}$ is set to 10, the SNR is maintained at 22 dB, and the number of Monte Carlo trials (*M*) is 50.

**Figure 6:**
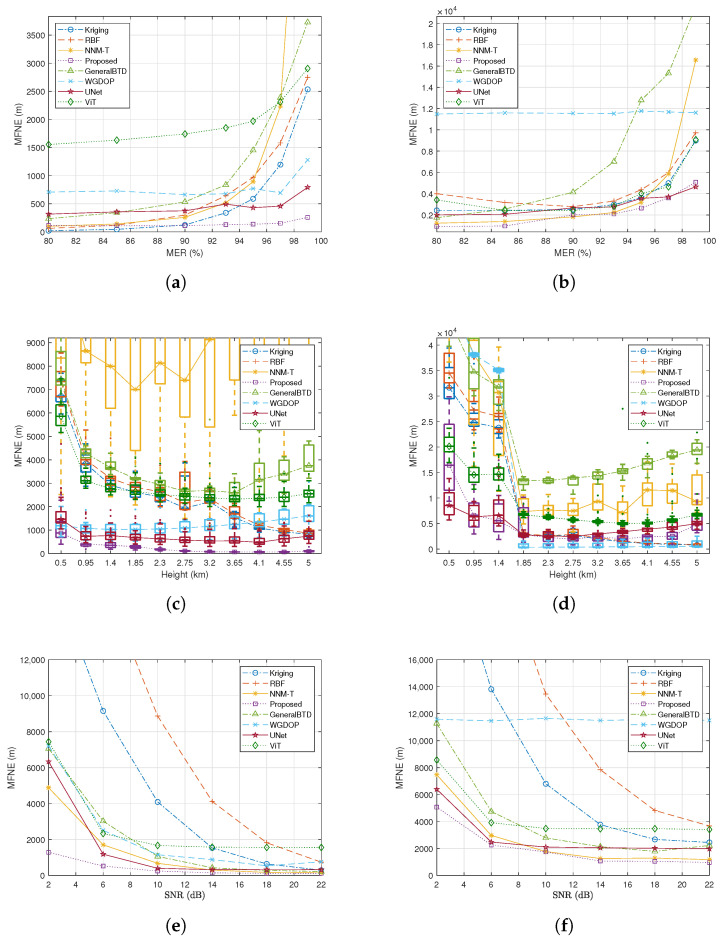
Comparison of reconstruction performance between the proposed algorithm and baseline methods ($f_{s}=10,M=50$). (**a**,**b**) Reconstruction MFNE versus MER at SNR=22 dB for $S_{\mathrm{TDOA}}$ and $S_{\mathrm{TDOA}}^{N}$. (**c**,**d**) Boxplots of reconstruction MFNE versus elevation with MER=99% (observation ratio = 1%) and SNR=22 dB for $S_{\mathrm{TDOA}}$ and $S_{\mathrm{TDOA}}^{N}$. (**e**,**f**) Reconstruction MFNE versus SNR with MER=80% (observation ratio = 20%) for $S_{\mathrm{TDOA}}$ and $S_{\mathrm{TDOA}}^{N}$.

Figure 6 compares the reconstruction performance of the proposed algorithm against several baseline methods across different MER levels. The baseline algorithms included in the comparison encompass both classic reconstruction algorithms and deep learning (DL)-based generative algorithms. It is worth noting that the scenario addressed in this paper is a “single-shot reconstruction” problem, characterized by extremely sparse valid observations and the absence of a historical database. Traditional DL methods typically fail in such zero-shot and extremely sparse conditions due to the inability to acquire sufficient training data. To enable the neural networks to function in this context, we generated 50 sets of historical data to serve as prior information for each training session. Although this constitutes an unfair comparison (as it provides an advantage to the DL baselines), we include these results to verify the superiority of the proposed algorithm from another perspective.

Figure 6a,b demonstrate that the proposed algorithm achieves the lowest MFNE, indicating superior reconstruction performance. Specifically, at a high sparsity level of MER=99% (only 1% observation ratio), the proposed algorithm exhibits a performance improvement ranging from 43.58% to 97.23% compared to the baseline methods, as detailed in Table 1. Here, Uplift(%) is defined as $\left({\mathrm{MFNE}}_{\mathrm{baseline}}-{\mathrm{MFNE}}_{\mathrm{proposed}}\right)/{\mathrm{MFNE}}_{\mathrm{baseline}}\times 100\%$. Note that the GeneralBTD method represents the standard BTD without the proposed physics-based polynomial constraints. The significant performance gap between the proposed algorithm and GeneralBTD quantifies the contribution of the proposed physical constraints. Furthermore, comparative analysis indicates that the proposed algorithm attains comparable reconstruction accuracy while requiring significantly fewer emitters. This substantial reduction in required emitters can translate to considerable operational cost savings.

Figure 6c,d present boxplots illustrating the distribution of MFNE at different elevations for MER=99%. As shown in Figure 6d, the introduction of NLOS error at altitudes between 0.5 km and 1.4 km leads to significant reconstruction errors for the WGDOP algorithm in this range. Consequently, its MFNE in Figure 6b is much higher than that of the other algorithms. These boxplots summarize key statistical measures such as quartiles and medians. A smaller inter-quartile range and more compact box shape generally indicate more consistent performance (i.e., higher robustness) across varying elevations. The results suggest that the proposed algorithm exhibits superior consistency compared to the baseline methods in this regard.

### 6.3. Performance Under Noise

In this subsection, we investigate the impact of observational noise on the algorithm’s reconstruction performance. We evaluate the reconstruction performance of the proposed and baseline schemes for SNR values ranging from 2 dB to 22 dB. For these experiments, the down-sampling factor $f_{s}$ was fixed at 10, the MER at 80%, and the number of Monte Carlo trials (*M*) at 50.

Figure 6e,f illustrate the influence of varying SNR levels on the reconstruction performance. Across the tested noise levels, the proposed algorithm consistently demonstrates superior reconstruction performance and exhibits greater resilience to noise compared to the other methods.

To quantitatively assess this noise resilience, we define a metric termed “Slope” as the average absolute rate of change of MFNE with respect to SNR:

(38) $$\mathrm{Slope}=\frac{1}{N_{\mathrm{snr}}-1}\sum_{n=1}^{N_{\mathrm{snr}}-1}\left|\frac{{\mathrm{MFNE}}_{n+1}-{\mathrm{MFNE}}_{n}}{{\mathrm{SNR}}_{n+1}-{\mathrm{SNR}}_{n}}\right|,$$

where ${\mathrm{SNR}}_{n}$ and ${\mathrm{MFNE}}_{n}$ represent the *n*-th smallest SNR value and its corresponding reconstruction error, respectively. $N_{\mathrm{snr}}$ denotes the total number of distinct SNR levels evaluated in the experiment. A smaller Slope value indicates better noise robustness. Table 2 presents the Slope values for all algorithms in both scenarios. The percentage improvement in this Slope metric for the proposed algorithm, relative to the baseline methods, ranges from 7.11% to 95.92%, indicating superior noise adaptability. In scenario $S_{\mathrm{TDOA}}^{N}$, the WGDOP method’s low Slope value is notable, primarily because NLOS errors, rather than observational noise, dictate its reconstruction accuracy. Figure 6d shows high reconstruction errors for this method at elevations from 0.5 km to 1.4 km due to multipath effects. Thus, despite good noise robustness, its overall reconstruction performance is substantially inferior to the proposed algorithm, corroborating the conclusions in Table 2.

### 6.4. Performance with Available Directional Measurements

In this subsection, we evaluate the proposed algorithm’s performance in a scenario where directional measurements of positioning errors are available. This validates the extended algorithm introduced in Section 5.2, assessing its reconstruction accuracy for both scalar ($\underline{G}$) and vector (${\underline{G}}_{r}$) representations of the spatial distribution of positioning error.

Table 3 presents performance metrics under varying degrees of MER. Here, ${\mathrm{MFNE}}_{1}$ quantifies the reconstruction error for the $\underline{G}$. ${\mathrm{MFNE}}_{2}$ is computed as the mean of the individual MFNEs obtained for each directional component tensor: ${\mathrm{MFNE}}_{2}=\frac{1}{3}\sum_{r=1}^{3}{\mathrm{MFNE}}_{{\underline{G}}_{r}}$. Comparing the results in Table 3 with those presented for the scalar error model in Figure 6a, it is found that incorporating directional information enhances the proposed algorithm’s performance. This improvement is observed in the reconstruction of both scalar and vector distributions of positioning errors across various MER conditions.

**Figure 7:**
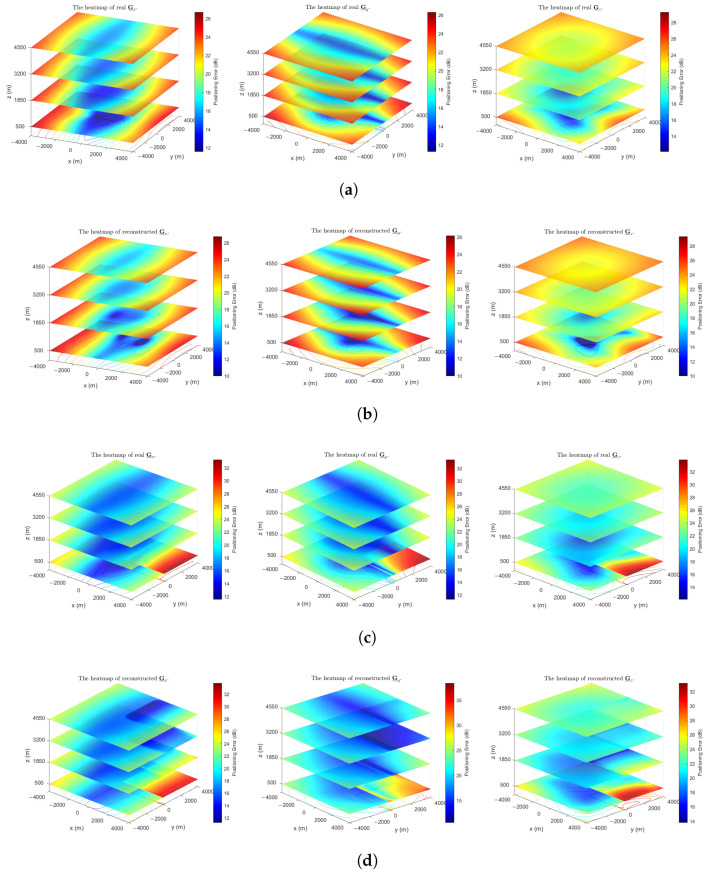
Heatmaps of real and reconstructed directional distributions of positioning error (MER=99%, $f_{s}=10$, SNR=22 dB). Values are shown on a dB scale for visualization. (**a**) The real heatmaps of positioning error distribution in $S_{\mathrm{TDOA}}$. (**b**) The reconstructed heatmaps of positioning error distribution in $S_{\mathrm{TDOA}}$. (**c**) The real heatmaps of positioning error distribution in $S_{\mathrm{TDOA}}^{N}$. (**d**) The reconstructed heatmaps of positioning error distribution in $S_{\mathrm{TDOA}}^{N}$.

To more intuitively demonstrate the reconstruction performance for ${\underline{G}}_{r}$, Figure 7 depicts heatmaps of the true and reconstructed directional component tensors in $S_{\mathrm{TDOA}}$ and $S_{\mathrm{TDOA}}^{N}$. For enhanced visualization, particularly when positioning errors are small, the heatmap values are presented on a dB scale, specifically showing $10\log_{10}\left({\underline{G}}_{r}\right)$ for r∈{1,2,3}.

### 6.5. Real-Data Experiment

**Figure 8:**
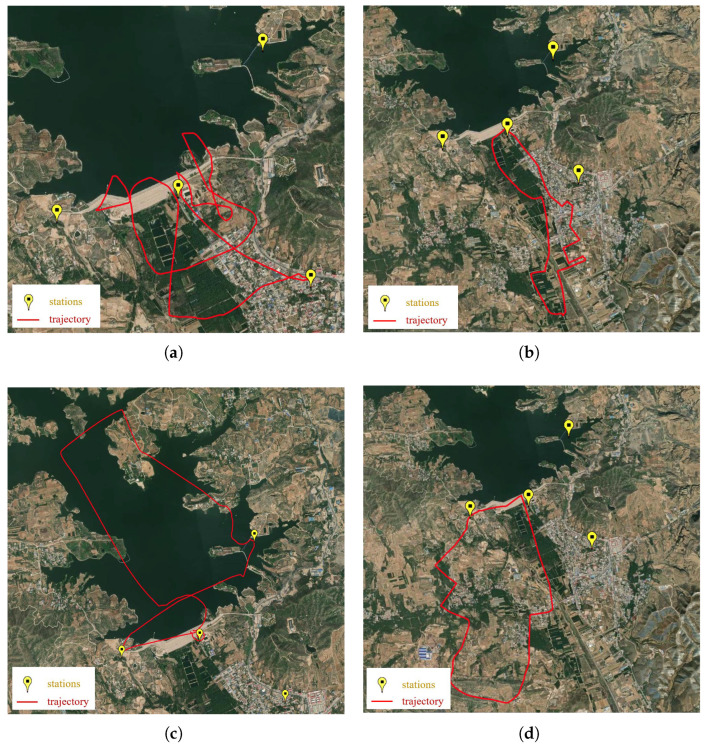
Schematic diagram of the experimental site, sensor stations layout, and UAV flight trajectories. (**a**) Trajectory $S_{1}$: flight trajectory in large-scale open terrain. (**b**) Trajectory $S_{2}$: flight trajectory in small-scale open terrain. (**c**) Trajectory $S_{3}$: low-altitude flight trajectory over a lake. (**d**) Trajectory $S_{4}$: low-altitude flight trajectory in an urban area.

In this subsection, we further validate the proposed algorithm using a real-world dataset collected from a TDOA positioning system. The dataset covers an area of 2500×4500 m^2^ and includes measurements across 120 frequency bands, ranging from 2.397 GHz to 2.519 GHz. The experimental setup comprises four sensors and an unmanned aerial vehicle (UAV) acting as the emitter. The sensors are deployed in a Y-shaped configuration, with baseline distances of 1 km, 1.1 km, and 1.3 km from the peripheral sensors to the central reference sensor, respectively. Figure 8 illustrates the experimental site, including the sensor layout and four sets of flight trajectories for the UAV.

A key challenge presented by this dataset is its extreme sparsity. For example, the trajectory $S_{4}$ is sampled at 386 distinct locations, with ground-truth positions from its onboard GPS. To analyze the spatial distribution of positioning errors, the target area was discretized into a fine-grained 200×200×10 grid. Consequently, the 386 measurement points represent only a tiny fraction of the 400,000 grid points, resulting in a MER of approximately 99.9%. This high-sparsity scenario serves as a stringent test for the algorithm’s tensor completion capabilities. To evaluate the algorithm’s performance under these conditions, we adopt a cross-validation approach. For each Monte Carlo trial, a subset of the observed data is randomly selected for training the algorithms, with the remainder reserved for testing. The proportion of data used for training is denoted by ρ. A total of M=50 Monte Carlo trials are conducted for each value of ρ.

Table 4 presents the percentage of performance improvement of the proposed algorithm compared with the comparative methods in various trajectories. The final column indicates whether an algorithm was capable of reconstructing the complete tensor (“Y” for yes, “N” for no), as some methods could only reconstruct partial slices or fail under extreme sparsity. The performance of the proposed algorithm is comparable to that of the classical GDOP method under simple channel conditions (such as $S_{2}$), while it has obvious advantages in complex channel environments (such as $S_{4}$). It can also be observed from Table 4 that under relatively ideal environmental conditions ($S_{2}$), the proposed algorithm yields only marginal performance improvements compared to simpler baseline algorithms (e.g., WGDOP), despite possessing significantly higher computational complexity. This raises the question of determining the appropriate scenarios for its application. In practice, however, error map reconstruction is typically performed offline to evaluate the station deployment of positioning systems; thus, time complexity is not the primary concern. In such cases, it is recommended to employ the proposed algorithm across all scenarios, trading higher computational complexity for a performance gain of approximately 30%. Conversely, for time-sensitive scenarios where computational efficiency is critical, we recommend restricting the use of the proposed algorithm to complex terrains, such as urban areas or canyons.

Then, we take trajectory $S_{4}$ as an example to give a further analysis. Table 5 presents the average MFNE of the reconstructed positioning error tensor on the test sets in trajectory $S_{4}$, along with computation times.

When comparing the proposed tensor-based modeling approach with WGDOP, we observe that our method further reduces the MFNE by over 27.96%. This highlights the benefit of our model in capturing complex error distributions. In contrast, traditional data-driven interpolation methods such as RBF and Kriging struggle significantly under such highly sparse conditions, particularly when entire rows or columns (or more generally, large contiguous regions) of the tensor lack measurements, leading to their failure in reconstructing the complete tensor. The NNM-T algorithm was also tested but is omitted from the table as it failed to converge reliably under this level of data scarcity. It also indicates that the proposed algorithm incurs a longer computation time compared to some baselines. However, real-time inference is often not a primary requirement because the analysis of the positioning error map is typically performed offline as a post-processing step.

**Figure 9:**
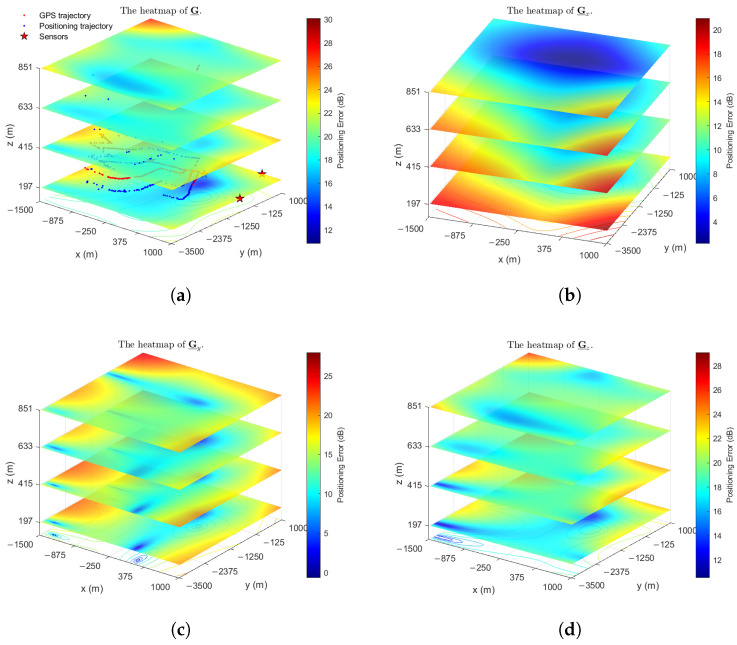
Reconstructed positioning error map for trajectory $S_{4}$ (ρ=80%, M=50). Values are presented on a dB scale for visualization. (**a**) Reconstructed heatmap of the spatial distribution of positioning error. (**b**–**d**) Reconstructed heatmaps of the spatial distribution of positioning error in x, y, and z directions, respectively.

Figure 9 presents heatmaps of the reconstructed positioning error map from the real-world data using the proposed algorithm. It suggests that the proposed method effectively models underlying error characteristics and can provide valuable guidance for system analysis. For instance, examining the magnitudes of the reconstructed directional error components in Figure 9b–d, we observe that the error magnitudes typically follow the relationship $G_{x}\geq G_{y}\geq G_{z}$. This implies that the positioning reliability is highest along the z-axis and lowest along the x-axis for this specific setup, an observation consistent with statistical analysis of the raw positioning data.

## 7. Conclusions

This paper tackles the critical challenge of high-fidelity reconstruction of spatial localization error maps from limited measurements. We propose a novel tensor-based framework that overcomes the limitations of conventional model-driven approaches, which are constrained by idealized assumptions, and purely data-driven methods that are dependent on dense observational data. Central to our approach is the TGDOP model, a novel representation that captures the complexity and anisotropic nature of localization errors. To address the practical issue of data scarcity, we developed a physics-informed tensor completion algorithm. By incorporating prior knowledge derived from the analytical properties of the error covariance matrix directly into the factorization process, our algorithm achieves robust reconstruction even from severely incomplete data. Comprehensive experiments on both simulated and real-world TDOA datasets demonstrate the superiority of the proposed method. Specifically, under extremely sparse conditions, our method improves reconstruction accuracy by at least 27.96% compared to state-of-the-art baselines. Future work will explore more efficient optimization strategies for large-scale scenarios and investigate advanced constraints on factor matrices to capture richer physical information.

## Figures and Tables

**Table 1:** Reconstruction Performance Comparison: $f_{s}=10$, SNR=22 dB, MER=99% (Observation Ratio = 1%), M=50.

Algorithm	Measure	$S_{\mathrm{TDOA}}$	$S_{\mathrm{TDOA}}^{N}$
Kriging [18]	MFNE	2534.48	8995.53
	Uplift (%)	89.85	43.58
RBF [20]	MFNE	2747.93	9727.88
	Uplift (%)	90.64	47.83
NNM-T [30]	MFNE	9301.32	16,570.32
	Uplift (%)	97.23	69.37
GeneralBTD [28]	MFNE	3728.42	21,644.72
	Uplift (%)	93.10	76.55
WGDOP [16]	MFNE	1279.25	11,614.96
	Uplift (%)	79.89	56.30
UNet [31]	MFNE	791.38	4646.17
	Uplift (%)	67.50	−9.24
ViT [33]	MFNE	2905.96	9074.46
	Uplift (%)	91.15	44.07
Proposed	MFNE	257.21	5075.51

**Table 2:** Noise Resistance Performance ($f_{s}=10$, MER=80%, M=50).

Algorithm	Measure	$S_{\mathrm{TDOA}}$	$S_{\mathrm{TDOA}}^{N}$
Kriging	Slope	845.03	1229.64
	Uplift (%)	93.03	83.30
RBF	Slope	1325.49	2136.86
	Uplift (%)	95.56	90.39
NNM-T	Slope	236.91	318.10
	Uplift (%)	75.15	35.46
GeneralBTD	Slope	342.55	496.45
	Uplift (%)	82.82	58.64
WGDOP	Slope	344.77	29.86
	Uplift (%)	82.93	−587.52
UNet	Slope	301.14	221.04
	Uplift (%)	80.45	7.11
ViT	Slope	294.38	259.26
	Uplift (%)	80.00	20.81
Proposed	Slope	58.87	205.32

**Table 3:** Performance with directional measurements for various MER ($f_{s}=10$, SNR=22 dB, M=50).

MER (%)	Measure	$S_{\mathrm{TDOA}}$	$S_{\mathrm{TDOA}}^{N}$
80	${\mathrm{MFNE}}_{1}$	138.34	1204.34
	${\mathrm{MFNE}}_{2}$	161.25	933.64
85	${\mathrm{MFNE}}_{1}$	133.66	897.13
	${\mathrm{MFNE}}_{2}$	160.90	827.88
90	${\mathrm{MFNE}}_{1}$	138.68	1089.64
	${\mathrm{MFNE}}_{2}$	162.45	892.21
95	${\mathrm{MFNE}}_{1}$	143.59	2463.44
	${\mathrm{MFNE}}_{2}$	170.82	1744.21
99	${\mathrm{MFNE}}_{1}$	223.47	3818.02
	${\mathrm{MFNE}}_{2}$	226.59	2639.67

**Table 4:** Performance improvement (%) of the Proposed Algorithm compared with the Comparison Algorithms in four trajectories.

Algorithm	Trajectories	Training Ratio (ρ)			Full Recon.
		40%	60%	80%	
WGDOP	$S_{1}$	1.02	9.70	13.37	Y
	$S_{2}$	−0.57	−0.88	3.19	
	$S_{3}$	24.72	26.35	19.63	
	$S_{4}$	27.96	39.42	30.64	
GeneralBTD	$S_{1}$	15.08	23.21	26.64	Y
	$S_{2}$	19.33	22.75	21.82	
	$S_{3}$	26.94	27.23	24.68	
	$S_{4}$	27.93	32.2	31.14	
Kriging	$S_{1}$	76.36	83.94	83.66	N
	$S_{2}$	57.65	50.11	55.77	
	$S_{3}$	63.32	56.96	33.35	
	$S_{4}$	62.37	72.94	67.95	
RBF	$S_{1}$	25.92	84.21	27.74	N
	$S_{2}$	28.69	25.54	24.56	
	$S_{3}$	21.21	14.07	10.68	
	$S_{4}$	28.12	32.57	33.01	

**Table 5:** Performance on Trajectory $S_{4}$, where MFNE is reported for the Test Set.

Algorithm	Measure	Training Ratio (ρ)			Full Recon.
		40%	60%	80%	
WGDOP	MFNE	968.81	888.10	493.45	Y
	Time (s)	9.53	9.85	9.79	
GeneralBTD	MFNE	968.39	793.58	497.02	Y
	Time (s)	80.22	83.55	83.42	
Kriging	MFNE	1854.52	1988.16	1067.84	N
	Time (s)	0.36	0.46	0.57	
RBF	MFNE	971.00	797.94	510.87	N
	Time (s)	0.04	0.06	0.07	
Proposed	MFNE	697.91	538.02	342.25	Y
	Time (s)	86.89	91.25	90.38	

## Data Availability

The codes and datasets in this paper are available at https://github.com/gitZHZhang/Code_and_Data_for_TGDOP.git (accessed on 7 January 2026).

## Appendix A. Proof of Theorem 1

Consider a 3-D scenario with a single emitter at a true location $u={\left[\mu_{x},\mu_{y},\mu_{z}\right]}^{T}$. We have *N* positioning results for this emitter, denoted as $u_{i}={\left[x_{i},y_{i},z_{i}\right]}^{T}$ for i=1,…,N. We assume the positioning errors are drawn from a distribution with mean 0 and a covariance matrix P, where the (j,k)-th element of P is estimated as $P\left(j,k\right)=\frac{1}{N}\sum_{i=1}^{N}\left(u_{i}\left(j\right)-u\left(j\right)\right)\left(u_{i}\left(k\right)-u\left(k\right)\right)$. The positioning results $u_{i}$ are assumed to follow a multivariate normal distribution, and their spatial spread often forms an ellipsoid. The probability density function (PDF) of $u_{i}$ is given by

(A1) $$\begin{array}{cc} \; & f\left(u_{i}\right)=\frac{1}{{\left(2\pi \right)}^{3/2}{\left|P\right|}^{1/2}}\exp\left(-\frac{1}{2}{\left(u_{i}-u\right)}^{T}P^{-1}\left(u_{i}-u\right)\right). \end{array}$$

According to the properties of the multivariate normal distribution, a 3-D random variable that follows a joint Gaussian distribution has Gaussian marginal and conditional distributions. Thus, we have $x_{i}∼N\left(\mu_{x},P_{11}\right)$, $y_{i}∼N\left(\mu_{y},P_{22}\right)$, and $z_{i}∼N\left(\mu_{z},P_{33}\right)$. To simplify the analysis and intuitively assess the error magnitudes along each coordinate axis, this paper utilizes the diagonal elements of the covariance matrix to approximately characterize the extent of the positioning error. So, we can treat $x_{i}$, $y_{i}$, and $z_{i}$ as independent variables in the following analysis.

Applying this to the positioning results $u_{i}$ relative to the true emitter location u, each component of the *i*-th positioning result follows $u_{i}\left(r\right)∼N\left(u\left(r\right),P\left(r,r\right)\right)$ for r∈{1,2,3} and i=1,…,N. Suppose that $\underline{\hat{G}}$ and ${\underline{\hat{G}}}_{r}$ are calculated from the *N* positioning results $u_{i}$ relative to the true location u. Since $\frac{u_{i}\left(r\right)-u\left(r\right)}{\sqrt{P(r,r)}}∼N\left(0,1\right)$, it follows that ${\left(\frac{u_{i}\left(r\right)-u\left(r\right)}{\sqrt{P(r,r)}}\right)}^{2}∼\chi^{2}\left(1\right)$ (Chi-squared distribution with 1 degree of freedom). Thus, ${\underline{\hat{G}}}_{r}$ can be written as

(A2) $$\begin{array}{cc} {\underline{\hat{G}}}_{r} & =\frac{P(r,r)}{N}\sum_{i=1}^{N}{\left(\frac{u_{i}\left(r\right)-u\left(r\right)}{\sqrt{P(r,r)}}\right)}^{2}\\ \; & ∼\frac{P(r,r)}{N}\chi^{2}\left(N\right)\equiv \frac{P(r,r)}{N}\mathrm{Gamma}\left(\frac{N}{2},\frac{1}{2}\right), \end{array}$$

where Gamma(α,β) denotes a Gamma distribution with shape α and rate β (or 1/β as scale). Here, α=N/2 and rate β=1/2.

The total tensor is decomposed into the sum of three terms, that is, $\underline{\hat{G}}=\sum_{r}{\underline{\hat{G}}}_{r}$. Note that ${\underline{\hat{G}}}_{1},{\underline{\hat{G}}}_{2},{\underline{\hat{G}}}_{3}$ are independent sums of squared normals, then the distribution of their sum $\underline{\hat{G}}$ would be a sum of scaled independent $\chi^{2}\left(N\right)$ variables, which generally has a complex form (generalized Chi-squared distribution).

However, by the Central Limit Theorem (CLT), for large *N*, each ${\underline{\hat{G}}}_{r}$ can be approximated by a Gaussian distribution: $E\left[{\underline{\hat{G}}}_{r}\right]=P\left(r,r\right)$ and $\mathrm{Var}\left({\underline{\hat{G}}}_{r}\right)=\frac{2P{\left(r,r\right)}^{2}}{N}$. Thus, for large *N*,

(A3) $$\begin{array}{cc} {\underline{\hat{G}}}_{r} & ∼N\left(P\left(r,r\right),\frac{2P{\left(r,r\right)}^{2}}{N}\right),\;\mathrm{for}\;r\in \left\{1,2,3\right\}. \end{array}$$

For the sum $\underline{\hat{G}}=\sum_{r}{\underline{\hat{G}}}_{r}$, $E\left[\underline{\hat{G}}\right]=\sum_{r}E\left[{\underline{\hat{G}}}_{r}\right]=\sum_{r}P\left(r,r\right)=\mathrm{tr}\left(P\right)$, and $\mathrm{Var}\left(\underline{\hat{G}}\right)=\sum_{r}\mathrm{Var}\left({\underline{\hat{G}}}_{r}\right)=\sum_{r}\frac{2P{\left(r,r\right)}^{2}}{N}$. Then, for large *N*,

(A4) $$\begin{array}{cc} \underline{\hat{G}} & ∼N\left(\mathrm{tr}\left(P\right),\sum_{r=1}^{3}\frac{2P{\left(r,r\right)}^{2}}{N}\right). \end{array}$$

Note that N, the number of samples, represents repeated localizations of an emitter at a specific position. Consequently, for a stationary emitter, acquiring a substantial sample size N is typically feasible in practical applications.

Correspondingly, the estimation errors ${\underline{N}}_{r}={\underline{\hat{G}}}_{r}-P\left(r,r\right)$ and $\underline{N}=\underline{\hat{G}}-\mathrm{tr}\left(P\right)$ follow

(A5) $$\begin{array}{cc} {\underline{N}}_{r} & ∼N\left(0,\frac{2P{\left(r,r\right)}^{2}}{N}\right),\;\mathrm{for}\;r\in \left\{1,2,3\right\},\\ \underline{N} & ∼N\left(0,\sum_{r\in \{1,2,3\}}\frac{2P{\left(r,r\right)}^{2}}{N}\right). \end{array}$$

## Appendix B. Proof of Theorem 2

Considering the emitter’s position $u={\left[x,y,z\right]}^{T}$ as the independent variable in a 3-D scenario, differentiating both sides of (15) with respect to u yields

(A6) $$\begin{array}{cc} \nabla_{u}P & =\left[\begin{array}{ccc} \nabla_{x}P & \nabla_{y}P & \nabla_{z}P \end{array}\right],\;\mathrm{where}\\ \nabla_{p}P & =\nabla_{p}C\tilde{P}C^{T}+C\tilde{P}\nabla_{p}C^{T}\;p\in \left\{x,y,z\right\}. \end{array}$$

To calculate the $\nabla_{p}C$, we obtain

(A7) $$\begin{array}{cc} \nabla_{p}C= & \underset{\mathrm{Term}\;1}{\underset{︸}{-{\left(F^{T}F\right)}^{-1}\left(F^{T}\nabla_{p}F+\nabla_{p}F^{T}F\right){\left(F^{T}F\right)}^{-1}F^{T}}}\\ + & \underset{\mathrm{Term}\;2}{\underset{︸}{{\left(F^{T}F\right)}^{-1}\nabla_{p}F^{T}}}. \end{array}$$

Then, we analyze the continuity of the two terms in $\nabla_{p}C$.

The continuity of Term 1 is contingent upon the following:

- Continuity of ${\left(F^{T}F\right)}^{-1}$: If $F\left(p\right)\in C^{1}\left(I\right)$ (i.e., F(p) is continuously differentiable on the interval *I*), then its transpose $F^{T}\left(p\right)$ is also $C^{1}\left(I\right)$. The product $F^{T}\left(p\right)F\left(p\right)$ is consequently $C^{1}\left(I\right)$. The matrix inversion operation, $A\mapsto A^{-1}$, is a smooth mapping on the set of invertible matrices. Therefore, provided $F^{T}\left(p\right)F\left(p\right)$ is invertible for all p∈I, ${\left(F^{T}\left(p\right)F\left(p\right)\right)}^{-1}$ is also $C^{1}\left(I\right)$.
- Continuity of product terms $F^{T}\nabla_{p}F$ and $\nabla_{p}F^{T}F$: Since $F\left(p\right)\in C^{1}\left(I\right)$, both F(p) and its derivative $\nabla_{p}F$ (and similarly $\nabla_{p}F^{T}$) are continuous on *I* (i.e., $\in C^{0}\left(I\right)$). Consequently, their products $F^{T}\left(p\right)\nabla_{p}F$ and $\nabla_{p}F^{T}F\left(p\right)$ are also continuous on *I*.

Given these points, Term 1, being composed of sums and products of continuous matrix functions (specifically, ${\left(F^{T}F\right)}^{-1}\in C^{1}\left(I\right)$, $F^{T}\in C^{1}\left(I\right)$, $\nabla_{p}F\in C^{0}\left(I\right)$, and $\nabla_{p}F^{T}\in C^{0}\left(I\right)$), is itself continuous on *I*. Then, we concentrate on the continuity of Term 2. As established, ${\left(F^{T}\left(p\right)F\left(p\right)\right)}^{-1}\in C^{1}\left(I\right)$ (and thus also $C^{0}\left(I\right)$). Since $F\left(p\right)\in C^{1}\left(I\right)$, its transpose derivative $\nabla_{p}F^{T}$ is continuous on *I* (i.e., $\in C^{0}\left(I\right)$). The product of these two continuous matrix functions, ${\left(F^{T}\left(p\right)F\left(p\right)\right)}^{-1}\nabla_{p}F^{T}$, is therefore continuous on *I*.

To sum up, a conclusion can be drawn that $\nabla_{p}P\in C^{0}\left(I\right)$ (that is, $P\in C^{1}\left(I\right)$ ) under the following conditions:

- $F\left(p\right)\in C^{1}\left(I\right)$.
- ∀p∈I, rank(F(p))=n (where *n* is the number of columns of F, ensuring $F^{T}F$ is invertible).

## Appendix C. Definition and Properties of a Tensor Operation

Analogous to how multiple systems of equations can be represented using matrix multiplication, we introduce a tensor operation, the mode-n transpose product, denoted as “$\times_{n}^{T}$”. This operation is designed to represent linear relationships between higher-order and lower-order tensors. A formal definition is provided below.

**Definition** **2** (Mode-n Transpose Product)**.** *Define a Kth-order tensor $\underline{X}\in R^{I_{1}\times \cdots \times I_{K}}$ and a cell array $U=\{U_{1},U_{2},\cdots ,U_{K}\}$. Here, $U_{n}$ is the weight matrix and the others are the mask matrices. The dimensions of these matrices are given by*

$$U_{i}\in \left\{\begin{array}{ccc} \; & R^{N_{i}\times N_{i}}\; & i\neq n\\ \; & R^{I_{n}\times 1}\; & i=n, \end{array}\right.$$

*where $N_{i}=\prod_{j=1,j\neq i}^{K-1}I_{v_{j}}$ and $v_{i}=\left\{\begin{array}{ccc} & i\; & i\neq \;n\\ & K\; & i=n \end{array}\right..$ Then, the mode-n transpose product of $\underline{X}$ and U is defined as*

(A8) $$\begin{array}{cc} \underline{Y}= & \underline{X}\times_{n}^{T}U,\\ {\underline{Y}}_{\left(i\right)}= & {\underline{X}}_{\left(v_{i}\right)}\;\cdot \;\left(U_{n}⊗U_{i}\right), \end{array}$$

*where*

$$\begin{array}{cc} U_{n}⊗U_{i} & \in R^{\left(I_{n}N_{i}\right)\times N_{i}}\;\left(i\neq n\right),\\ \underline{Y} & \in R^{I_{v_{1}}\times I_{v_{2}}\cdots \times I_{v_{K-1}}}. \end{array}$$

Compared with the mode-n product defined in (8), the mode-n product typically applies a linear transformation to the mode-n fibers without altering their fundamental vector structure. In contrast, the mode-n transpose product effectively compresses the tensor along its *n*-th dimension. Specifically, each mode-n fiber, $f_{n}=\underline{X}\left(i_{1},\ldots ,i_{n-1},:,i_{n+1},\ldots ,i_{K}\right)$, is mapped to a scalar value in the resulting tensor $\underline{Y}$.

Using the operator “$\times_{n}^{T}$”, (12) can be expressed as

(A9) $$\begin{array}{c} \underline{G}=\underline{X}\times_{4}^{T}\left\{I_{N_{2}N_{3}},I_{N_{1}N_{3}},I_{N_{1}N_{2}},1_{3}\right\}. \end{array}$$

Let us elaborate on this example to illustrate the fiber reorganization. We begin by noting the dimensions: $\underline{X}\in R^{N_{1}\times N_{2}\times N_{3}\times 3}$, $U_{4}\in R^{3\times 1}$, $U_{1}\in R^{N_{2}N_{3}\times N_{2}N_{3}}$, $U_{2}\in R^{N_{1}N_{3}\times N_{1}N_{3}}$, and $U_{3}\in R^{N_{1}N_{2}\times N_{1}N_{2}}$. According to (A8), we obtain

(A10) $$\begin{array}{cc} {\underline{G}}_{\left(1\right)}= & {\underline{X}}_{\left(1\right)}\;\cdot \;\left(1_{3}⊗I_{N_{2}N_{3}}\right), \end{array}$$

(A11) $$\begin{array}{cc} {\underline{G}}_{\left(2\right)}= & {\underline{X}}_{\left(2\right)}\;\cdot \;\left(1_{3}⊗I_{N_{1}N_{3}}\right), \end{array}$$

(A12) $$\begin{array}{cc} {\underline{G}}_{\left(3\right)}= & {\underline{X}}_{\left(3\right)}\;\cdot \;\left(1_{3}⊗I_{N_{1}N_{2}}\right). \end{array}$$

Suppose the fiber $\underline{X}\left(:,j,k,l\right)$ corresponds to the mth column in ${\underline{X}}_{\left(1\right)}$, and $\underline{X}\left(:,j+\Delta j,k+\Delta k,l+\Delta l\right)$ corresponds to the n-th column. Based on (6), we have

(A13) $$\begin{array}{cc} n= & m+\Delta j+\Delta k\;\cdot \;N_{2}+\Delta l\;\cdot \;N_{2}N_{3}. \end{array}$$

In the matrix $1_{3}⊗I_{N_{2}N_{3}}$, each pair of non-zero elements in the column vectors are separated by $N_{2}N_{3}$ zeros. Correspondingly, when Δj=0, Δk=0, and Δl=1 in (A13), the difference in the fiber column indices in ${\underline{X}}_{\left(1\right)}$ is $N_{2}N_{3}$. According to the rules of matrix operations, (A10) is equivalent to

(A14) $$\begin{array}{cc} \underline{G}\left(:,j,k\right)= & \sum_{l=1}^{3}\underline{X}\left(:,j,k,l\right). \end{array}$$

Similarly, analogous analysis results can be derived for Equations (A11) and (A12), that is,

(A15) $$\begin{array}{cc} \underline{G}\left(i,:,k\right)= & \sum_{l=1}^{3}\underline{X}\left(i,:,k,l\right),\\ \underline{G}\left(i,j,:\right)= & \sum_{l=1}^{3}\underline{X}\left(i,j,:,l\right). \end{array}$$

Next, we introduce the general physical meaning of the mode-n transpose product. Substitute $U_{4}={\left[\lambda_{1},\lambda_{2},\lambda_{3}\right]}^{T}$ in (A8). Reorganize the ${\underline{X}}_{\left(1\right)}$ into a block matrix form such that ${\underline{X}}_{\left(1\right)}=\left[\begin{array}{c} S_{\left(1,1\right)},S_{\left(2,1\right)},S_{\left(3,1\right)} \end{array}\right]$, where $S_{\left(i,j\right)}=\underline{X}{\left(:,:,:,i\right)}_{\left(j\right)}$, then we have

(A16) $$\begin{array}{cc} {\underline{G}}_{\left(1\right)}= & {\underline{X}}_{\left(1\right)}\;\cdot \;\left(U_{4}⊗U_{1}\right)\\ = & \sum_{i=1}^{3}\lambda_{i}\;\cdot \;S_{\left(i,1\right)}\;\cdot \;U_{1}. \end{array}$$

From (A16), we can conclude that the elements in $U_{4}$ are the weights of $S_{\left(i,1\right)}$ (i.e., the mode-1 unfolding matrices of the sub-tensors), and $U_{1}$ is the linear transformation matrix of $S_{i}$. Therefore, the mode-n transpose product is essentially a vectorized representation of linear operations on the sub-tensor slices of the tensor along the nth dimension. The weight matrix and the mask matrices play the same role in the matrices ${\underline{X}}_{\left(2\right)}$ and ${\underline{X}}_{\left(3\right)}$, so we could generalize (A16) as

(A17) $$\begin{array}{cc} {\underline{G}}_{\left(j\right)}= & \sum_{i=1}^{3}\lambda_{i}\;\cdot \;S_{\left(i,j\right)}\;\cdot \;U_{j}. \end{array}$$

## Appendix D. Computational Complexity Analysis

We analyze the computational complexity of the proposed algorithm per iteration. Let |Ω| denote the number of sparse observation samples, *R* be the number of block terms, and $\left(M_{1},M_{2},M_{3}\right)$ represent the multilinear ranks for the three modes, respectively. Additionally, let *m* denote the degree of the polynomial basis functions used in the physics-informed constraints. The algorithm employs a BCD strategy, which iteratively updates the core tensors and the factor matrices. The complexity is determined by the least squares (LS) solvers applied in each step. In the step of updating the core tensors ${\left\{S_{r}\right\}}_{r=1}^{R}$, the algorithm solves for all elements within the core tensors simultaneously. The total number of optimization variables, denoted as $P_{\mathrm{core}}$, is given by the sum of elements in *R* tensors of size $M_{1}\times M_{2}\times M_{3}$:

(A18) $$P_{\mathrm{core}}=R\;\cdot \;\left(M_{1}M_{2}M_{3}\right).$$

The update involves solving a linear least squares problem with |Ω| equations. The computational cost is dominated by the complexity of the LS solver, which is generally quadratic in the number of variables for a fixed number of observations:

(A19) $$C_{\mathrm{core}}\approx O\left(|\Omega |\;\cdot \;P_{\mathrm{core}}^{2}\right)=O\left(|\Omega |\;\cdot \;R^{2}{\left(M_{1}M_{2}M_{3}\right)}^{2}\right).$$

When updating the factor matrices like $V_{1}$ for mode-1 with polynomial constraints, the optimization targets the polynomial coefficient matrix $\Phi_{1}\in R^{m\times M_{1}}$ rather than the full factor matrix. The number of variables for mode-1 across *R* components is

(A20) $$P_{\mathrm{factor}}^{\left(1\right)}=R\;\cdot \;M_{1}\;\cdot \;m.$$

Consequently, the complexity for updating the factor matrices along all three modes is

(A21) $$C_{\mathrm{factor}}\approx \sum_{k=1}^{3}O\left(|\Omega |\;\cdot \;{\left(RM_{k}m\right)}^{2}\right)=O\left(\left|\Omega \right|R^{2}m^{2}\left(M_{1}^{2}+M_{2}^{2}+M_{3}^{2}\right)\right).$$

Combining the costs from the core tensor and factor matrix updates, the total computational complexity per iteration is

(A22) $$C_{\mathrm{total}}=O\left(\left|\Omega \right|R^{2}\left[{\left(M_{1}M_{2}M_{3}\right)}^{2}+m^{2}\left(M_{1}^{2}+M_{2}^{2}+M_{3}^{2}\right)\right]\right).$$

It is observed that the complexity term associated with the core tensors, ${\left(M_{1}M_{2}M_{3}\right)}^{2}$, grows with the product of the ranks across all modes, whereas the term associated with the factor matrices, $m^{2}M_{k}^{2}$, grows linearly with respect to the mode dimensions. In practical settings, the polynomial degree *m* is a small constant (typically $m\ll M_{1}M_{2}M_{3}$). Therefore, the core tensor update dominates the computational load:

(A23) $${\left(M_{1}M_{2}M_{3}\right)}^{2}\gg m^{2}\left(M_{1}^{2}+M_{2}^{2}+M_{3}^{2}\right).$$

As a result, the term involving *m* is often negligible in the asymptotic analysis, and the overall complexity can be simplified to

(A24) $$C_{\mathrm{total}}\approx O\left(|\Omega |\;\cdot \;R^{2}{\left(M_{1}M_{2}M_{3}\right)}^{2}\right).$$

## Appendix E. Proof of Theorem 4

Let the positioning error distribution in 3-D space be described by a function $f:\Omega \subset R^{3}\to R$. We construct the error tensor $G\in R^{I\times J\times K}$, where the elements are obtained by sampling on a spatial grid $\left(x_{i},y_{j},z_{k}\right)$:

(A25) $$G_{ijk}=f\left(x_{i},y_{j},z_{k}\right).$$

According to the high-dimensional generalization of the Schmidt decomposition, the low-rank property implies strong separability. We can approximate *f* as a sum of products of separable univariate functions:

(A26) $$f\left(x,y,z\right)\approx \sum_{p=1}^{P}\sum_{q=1}^{Q}\sum_{r=1}^{R}g_{pqr}\;\cdot \;\phi_{p}\left(x\right)\;\cdot \;\psi_{q}\left(y\right)\;\cdot \;\zeta_{r}\left(z\right),$$

where $g_{pqr}$ are the weighted coefficients, and $\left\{\phi_{p}\left(x\right)\right\},\left\{\psi_{q}\left(y\right)\right\},\left\{\zeta_{r}\left(z\right)\right\}$ are the principal component basis functions along the x,y,z axes, respectively. Since the original function *f* is $C^{1}$ continuous with respect to position, and the error field is typically governed by physical laws, integral operator theory dictates that the decomposed basis functions (e.g., $\phi_{p}\left(x\right)$) must inherit the smoothness of *f*. Thus, $\phi_{p}\left(x\right)\in C^{1}$. Consider an arbitrary basis function in the *x*-direction, $\phi_{p}\left(x\right)$. Since $\phi_{p}\left(x\right)$ is a $C^{1}$ continuous function defined on a closed interval, the Weierstrass Approximation Theorem is applicable. For a smooth function that is determined by the low-rank principal components, we can approximate it with high precision using a low-order polynomial $P_{p}\left(x\right)$ of degree *d*:

(A27) $$\phi_{p}\left(x\right)\approx \sum_{n=0}^{d}w_{p,n}x^{n}=w_{p,0}+w_{p,1}x+\cdots +w_{p,d}x^{d}$$

This implies that the function $\phi_{p}\left(x\right)$ approximately belongs to the polynomial function space spanned by $\left\{1,x,x^{2},\ldots ,x^{d}\right\}$. Returning to the discrete decomposition of tensor G:

(A28) $$G\approx S\times_{1}U\times_{2}V\times_{3}W\Leftrightarrow G_{xyz}\approx \sum_{p=1}^{P}\sum_{q=1}^{Q}\sum_{r=1}^{R}S_{pqr}u_{xp}v_{yq}w_{zr}.$$

Physically, each column $u_{p}$ of the mode-1 factor matrix $U\in R^{I\times P}$ represents the discretized vector of the basis function $\phi_{p}\left(x\right)$ evaluated at the sampling points $x_{1},\ldots ,x_{I}$:

(A29) $$u_{p}={\left[\begin{array}{cccc} \phi_{p}\left(x_{1}\right) & \phi_{p}\left(x_{2}\right) & \cdots & \phi_{p}\left(x_{I}\right) \end{array}\right]}^{T}$$

Combining this with the polynomial form in (A27), we expand $u_{p}$ as

(A30) $$u_{p}\approx \sum_{n=0}^{d}w_{p,n}{\left[\begin{array}{cccc} x_{1}^{n} & x_{2}^{n} & \cdots & x_{I}^{n} \end{array}\right]}^{T}$$

Define the matrix $D_{x}=\left[1,x,x^{2},\ldots ,x^{d}\right]\in R^{I\times (d+1)}$, the column vector $u_{p}$ of the factor matrix can be expressed as $u_{p}\approx D_{x}\;\cdot \;w_{p}$ Through the derivation above, we have proven that the column vectors of the factor matrix U (and similarly for V,W) can be approximated as linear combinations of the columns of a Vandermonde matrix. Therefore, the column vectors of the factor matrices lie within the linear subspace generated by the low-order polynomial basis $\left\{1,x,\ldots ,x^{d}\right\}$, that is,

(A31) $$\mathrm{span}\left(U\right)\subseteq \mathrm{span}(\left\{1,x,\ldots ,x^{d}\right\})$$
